# Consequences of domestication in eastern oyster: Insights from whole genomic analyses

**DOI:** 10.1111/eva.13710

**Published:** 2024-05-29

**Authors:** Honggang Zhao, Ximing Guo, Wenlu Wang, Zhenwei Wang, Paul Rawson, Ami Wilbur, Matthew Hare

**Affiliations:** ^1^ Department of Natural Resources & the Environment Cornell University Ithaca New York USA; ^2^ Haskin Shellfish Research Laboratory Rutgers University Port Norris New Jersey USA; ^3^ Department of Computer Sciences Texas A&M University‐Corpus Christi Corpus Christi Texas USA; ^4^ School of Marine Sciences University of Maine Orono Maine USA; ^5^ Shellfish Research Hatchery, Center for Marine Science University of North Carolina Wilmington Wilmington North Carolina USA; ^6^ Present address: Center for Aquaculture Technology San Diego California USA

**Keywords:** aquaculture, captivity, domestication, evolution, inbreeding, oyster

## Abstract

Selective breeding for production traits has yielded relatively rapid successes with high‐fecundity aquaculture species. Discovering the genetic changes associated with selection is an important goal for understanding adaptation and can also facilitate better predictions about the likely fitness of selected strains if they escape aquaculture farms. Here, we hypothesize domestication as a genetic change induced by inadvertent selection in culture. Our premise is that standardized culture protocols generate parallel domestication effects across independent strains. Using eastern oyster as a model and a newly developed 600K SNP array, this study tested for parallel domestication effects in multiple independent selection lines compared with their progenitor wild populations. A single contrast was made between pooled selected strains (1–17 generations in culture) and all wild progenitor samples combined. Population structure analysis indicated rank order levels of differentiation as [wild − wild] < [wild − cultured] < [cultured − cultured]. A genome scan for parallel adaptation to the captive environment applied two methodologically distinct outlier tests to the wild versus selected strain contrast and identified a total of 1174 candidate SNPs. Contrasting wild versus selected strains revealed the early evolutionary consequences of domestication in terms of genomic differentiation, standing genetic diversity, effective population size, relatedness, runs of homozygosity profiles, and genome‐wide linkage disequilibrium patterns. Random Forest was used to identify 37 outlier SNPs that had the greatest discriminatory power between bulked wild and selected oysters. The outlier SNPs were in genes enriched for cytoskeletal functions, hinting at possible traits under inadvertent selection during larval culture or pediveliger setting at high density. This study documents rapid genomic changes stemming from hatchery‐based cultivation of eastern oysters, identifies candidate loci responding to domestication in parallel among independent aquaculture strains, and provides potentially useful genomic resources for monitoring interbreeding between farm and wild oysters.

## 1 INTRODUCTION

Selective breeding and domestication involve both intentional artificial selection for “improved” trait values as well as adaptation by natural selection to the captive or rearing environment, both achieved through a combination of genomic and environmentally induced changes across and within generations, respectively (Price, 1984). The contexts for domestication are diverse, from food and fiber production to recreational and working pets. Equally or perhaps more diverse are contexts where captive breeding and propagation are needed for a species, but no genotypic or phenotypic change is desired. These include population supplementation and restoration, zoos, endangered species reintroductions, sterile insect releases, and maintenance of stocks for ecotoxicology, where test subjects are assumed to be representative of wild taxa (Brown et al., 2009; Frankham, 2008; Gaire et al., 2022). It is widely recognized that genetic changes are difficult to avoid in captivity, but directional changes caused by inadvertent natural selection in culture or as a result of rearing environment have been studied in relatively few taxa (Hoffmann & Ross, 2018). One well‐studied example is the Queensland fruit fly, bred in captivity across multiple generations to accumulate enough individuals for invasive control by releasing sterile males (Hendrichs et al., 2002). High mating success relative to wild males is required for sterile males to have a demographic effect, so the goal in captivity is to minimize evolutionary change. Nonetheless, comparison of relatively young (25 gen.) versus old (50 gen.) stocks showed the latter to have lower egg hatchability, shorter developmental time, higher fecundity, higher survival under stress, and greater longevity (Gaire et al., 2022). Measuring and understanding natural selection in culture can help anticipate and manage its effects in breeding and propagation contexts where evolutionary stasis is the goal.

Given that very strong artificial selection can be imposed on commercially valuable traits, it might seem intuitive that this intentional aspect of domestication is the primary driver for rapid evolutionary change. However, for species with high fecundity and high early mortality (type III survivorship curve), culture and rearing conditions can impose strong selection pressures that greatly impact relative fitness in terms of survival. For example, agricultural practices tend to increase intraspecific competition while reducing interspecific competition. Type III survivorship is particularly common among marine fishes and invertebrates and therefore very relevant to domestication for aquaculture (Hedgecock & Pudovkin, 2011). The early life stages of aquatic species are typically cultured in tanks at relatively high density, with ad libitum feeding and nutritionally homogeneous food relative to the wild. Some practices, such as deliberately culling runts to reduce density or size‐selective culling as a consequence of culture cleaning, generate predictable selection pressures. For the most part, selection imposed by rearing/culture conditions is inadvertent, unmeasured, but not necessarily weak.

Bivalve production via aquaculture has expanded rapidly in the last two decades, with an annual growth rate of 3.5% between 2000 and 2017 (Naylor et al., 2021). Despite the key roles of bivalves in supporting environmentally friendly marine aquaculture and human demand for animal protein, production gains through bivalve breeding programs are generally lagging behind some finfish and crustacean species (Houston et al., 2022). On the other hand, increases in yield and performance from selective breeding have been demonstrated empirically in bivalve species like the eastern oyster (Ford & Haskin, 1987; Guo, 2021; Haley, 1976; Proestou et al., 2016), the Pacific oyster (Degremont et al., 2015; Hershberger et al., 1984; Langdon et al., 2003), and the European flat oyster (Newkirk & Haley, 1983). Because many commercially valuable traits cannot be measured noninvasively for selection in bivalves, the trend toward the application of genetic tools provides welcome efficiencies in genomic selection (Guo et al., 2023). Also, long‐term sustainability requires genomic management to minimize inbreeding and mate allocation to maximize genomic diversity (Zenger et al., 2019). Thus, a better understanding of genomic variation and the changes accompanying rapid phenotypic responses to domestication selection can inform genomic‐based improvement programs.

In this study, we use “domestication” and “domestication selection” to specifically refer to selection by rearing methods/culture environment. We acknowledge that this distinction is not always clear in practice for some traits, but proceed with the rationale that directed artificial selection is likely to have different targets, or be applied somewhat differently and in different environments across breeding programs for a given species, whereas rearing method/culture conditions often conform to relatively uniform “best practices,” at least for early life stages when viability selection might be strongest and are applied within a relatively uniform environment across hatcheries. These standard hatchery practices were described in detail by Loosanoff and Davis (1963; the “Milford method”) and Castagna et al. (1996); that is, they have been similar for the duration of breeding history for existing lines. Therefore, genomic domestication effects found in common across selection lines are more likely related to natural selection during rearing/culture but may include ubiquitous selection targets like fast growth. The shared domestication effects we are concerned with occur across breeding lines within a species, not to be confused with “domestication syndromes” recognized by convergence across species (Wilkins et al., 2014).

For species with high fecundity and high early mortality, it is expected that strong selection can greatly impact the survival and reproductive performance of captive populations as a consequence of domestication, even after one generation in the artificial environment (Araki et al., 2008). This is important in the context of hatchery‐based population supplementation or restoration programs (Bersoza Hernández et al., 2018), and empirical evidence suggests that gene pool modification in domesticated populations may lead to reduced fitness once released into natural environments (Araki et al., 2007; Vincent, 1960). Adaptation to captivity is well documented in salmon, where a relatively homogeneous captive environment can increase the efficacy of selection pressures resulting from high population density, release from predation, and abundant nutritious food (Fleming & Einum, 1997; Wessel et al., 2006; Zhang et al., 2016). Salmon traits hypothesized to evolve in response to culture conditions include fast growth (Fleming et al., 2002), a reduced antipredator behavior (Jackson & Brown, 2011), and reduction in eye size (Perry et al., 2021). These domestication traits could be maladaptive under natural conditions (e.g., reduced eye size in the captive condition is a maladaptive trait selected against in the wild) (Perry et al., 2021).

Notwithstanding examples of captivity‐induced genetic and phenotypic changes, the genetic architecture underlying domestication traits is not well understood. Generally, sharing of genomic outliers across independent breeding lines, as predicted by the parallel evolution hypothesis, has infrequently been found when comparing multiple wild versus selected strains (López et al., 2019; Mäkinen et al., 2015; Vasemägi et al., 2012). This paucity of parallel domestication effects in genomes has been attributed to likely polygenic architectures for relevant traits. An alternative contributing factor could be linked selection in breeding lines with elevated linkage disequilibrium (LD), such that identified candidate loci are linked markers rather than the true target of parallel selection. Differing LD patterns across breeding lines could lead to distinct linked markers showing selection. Compared to domesticated fish species, little is known about the effects of domestication selection on bivalve organisms. Recent work in eastern oysters revealed that larval starvation tolerance may be a trait subject to domestication selection. In this case, domestication is hypothesized to entail a release from selection for the starvation tolerance that is believed to be important for larvae in nature (McFarland et al., 2020). Larval fitness under variable salinity and setting success under hatchery conditions are additional traits that have been inadvertently altered by domestication or selection for fast growth and disease resistance in the eastern oyster (McDonald et al., 2023). Similarly, we hypothesize that the culture environment required for selective breeding of high‐fecundity organisms like oysters imposes strong domestication selection on multiple traits that may be phenotypically cryptic. Examples of hatchery conditions that differ strongly from the wild include high gamete density in artificial crosses, higher larval culture densities, selection against smaller size classes (Taris et al., 2007), and/or truncation of size classes used for settlement (Nascimento‐Schulze et al., 2021). Genomic scans between wild and selected oysters may uncover genomic signatures that are relevant to domestication selection.

Processes of domestication and selective breeding will impose a number of evolutionary effects on domesticated species, and the extent of genetic changes to captivity depends upon the intensity of selection, effective population size (*N*_e_), genetic diversity, trait genomic architecture, control of inbreeding, and number of generations in captivity, as predicted by quantitative genetic theory (Frankham, 2008; Hill & Kirkpatrick, 2010). For example, empirical evidence has shown that domestication typically involves serial genetic bottlenecks and a continual low level of effective population size (*N*_e_). Populations with small values of *N*_e_ are relatively more prone to genetic drift and therefore lose genetic diversity more rapidly than high *N*_e_ populations. For example, in coho salmon the observed nonlinear pattern of *N*_e_ change over time in commercial and hatchery strains reflects a complex breeding history (Martinez et al., 2022). Experiments comparing effective population size between wild and hatchery strains of coho salmon, have found a significant decrease of *N*_e_ in hatchery and commercial strains within the last twenty generations (Martinez et al., 2022). The same trend of reduced effective population size was also reported across bivalve species when they were subject to captive breeding, including Pacific oysters (Hedgecock & Sly, 1990), pearl oysters (Lind et al., 2009), and abalone (Rhode et al., 2012). When breeding line *N*_e_ is reduced, it is associated with elevated linkage disequilibrium from genetic drift. Higher LD can make genomic selection more efficient because there is a stronger correlation between assayed markers and targets of selection (Wientjes et al., 2013). Similarly, more extensive LD can increase the power to identify a QTL or find genome‐wide associations with a phenotype (Hong & Park, 2012), but it can make it more difficult to identify causative variants within QTLs.

One of the most commonly reported genetic repercussions of selective breeding is the loss of genetic diversity, for example, as measured by heterozygosity or allele diversity. The decline in genetic diversity has been widely reported in aquatic species undergoing domestication and mass selection, including finfish (López et al., 2019; Sawayama & Takagi, 2016) and mollusks (Chen et al., 2017; Lind et al., 2009), including the eastern oyster (Yu & Guo, 2005). Reduced genetic variation can increase the risk of inbreeding depression, constrain the genetic gains from selective breeding, and ultimately decrease the performance and fitness of captive populations (Xu et al., 2019). These empirical observations of genetic variability in aquaculture species could be related to the number of founder individuals, hatchery or spawning practices, or strategies of broodstock maintenance (e.g., periodic wild individual breeding inputs to increase genetic diversity).

A major concern of breeding programs is the presence of inbreeding and the potential to trigger inbreeding depression in captive populations (Araki et al., 2008; Frankham, 2008). Inbreeding depression refers to the reduction in mean fitness of a population due to increased homozygosity arising from the breeding of related individuals (Ceballos et al., 2018; Charlesworth & Charlesworth, 1987, 1990). There are multiple mechanisms that can contribute to inbreeding depression, but the increasing homozygosity of deleterious recessive alleles caused by inbreeding is the major cause (Charlesworth & Willis, 2009). Species with a high genetic load of deleterious recessive alleles are expected to suffer greater inbreeding depression for a given level of inbreeding. Highly fecund organisms are predicted to have a high genetic load resulting from mutations during many germ‐cell divisions (Williams, 1975), and a high genetic load has been reported in many species with this life history (Plough, 2016), including oysters (Launey & Hedgecock, 2001; Plough et al., 2016). For shellfish, the deleterious effects of inbreeding have been well documented when animals were kept in captivity with small effective population sizes (Evans et al., 2004; Vendrami et al., 2019). Some inbreeding is unavoidable in a breeding line, but it can be minimized to perpetuate artificial selection responses by applying moderate selection intensity and maintaining an adequately large pool of broodstock (Evans et al., 2004). Selective breeding may also purge recessive lethal alleles over time and reduce genetic load or inbreeding depression (Yu & Guo, 2003).

Inbreeding gives rise to uninterrupted long runs of homozygosity (ROH) that are identical‐by‐descent (IBD; Ceballos et al., 2018). The genome inbreeding coefficient based on runs of homozygosity (F_ROH_) is a superior and accurate metric for individual inbreeding as it does not rely on allele frequencies or sampling procedures (Ceballos et al., 2018; Talebi et al., 2020). Populations with different demographic histories can harbor divergent distributions of ROH, with longer ROH segments originating from the mating of relatives with recent common ancestors and shorter segments resulting from background relatedness during the more extended population history (Ceballos et al., 2018). A fine‐scale investigation of ROH distribution can reveal recombination hotspots (also known as ROH islands) or coldspots along the genome, potentially illuminating the demographic history of the population and selection signatures related to domestication processes (Ceballos et al., 2018). The genetic architecture of ROH has been well described in mammals and plants but is less studied in aquatic species, especially bivalves. By quantifying and comparing the ROH segments within wild and selected strain oysters, we are able to estimate the inbreeding coefficient and identify homozygous genomic regions that may be responsible for inbreeding depression, potentially enabling the identification of mechanisms contributing to performance differences.

In this study, we take advantage of recently developed genomic resources in the eastern oyster, *Crassostrea virginica*, to measure genetic changes resulting from selective breeding and test for parallel culture‐induced domestication effects. The eastern oyster is an important aquaculture species on the East Coast of the United States and has been selectively bred for only a few decades (since 1960). Natural populations of eastern oysters are only a fraction of their historical density because of overfishing, habitat destruction, and disease prevalence (Beck et al., 2011; Powell et al., 2008; Schulte, 2017; Zu Ermgassen et al., 2012). Growing interest in aquaculture motivated the selective breeding of strains with commercially valuable traits. The extent and history of eastern oyster domestication efforts vary by geographic region, historically involving mass selection via exposure to natural disease epizootics or mass selection for commercially valuable traits such as shell shape, coloration, and faster growth. For instance, during the early 1960s, intensive selection efforts were applied to the eastern oyster to produce lines resistant to the multinucleated sphere unknown (MSX) disease caused by the protist *Haplosporidium nelsoni* (Haskin & Ford, 1979). Subsequent development of oyster lines resistant to MSX has increased survival 8‐fold and demonstrated that disease‐resistance traits could significantly respond to selection within a few generations (Ford & Haskin, 1987; Haskin & Ford, 1979). Since then, a variety of eastern oyster selected lines have been developed for research and industry use, including the Northeast High Survival line (NEH), which was initially selected for MSX and Dermo disease resistance and fast growth (Guo, 2021; Yu & Guo, 2006), and the University of Maine Flowers Select (UMFS) line, which was selected for improved cold water growth performance and resistance to Roseovarius oyster disease (ROD; Rawson & Feindel, 2012). In addition, a DBX strain and multiple sublines were developed in Delaware Bay (DB) with faster adult growth and disease resistance (McDonald et al., 2023).

Using the eastern oyster as an example, this study seeks to characterize genomic changes between multiple selected strains and their progenitor wild populations. A single contrast was made between pooled selected strains and pooled natural populations from which strains were originally sourced to examine these independent transitions. Genetic diversity metrics, coupled with linkage disequilibrium and runs of homozygosity, were quantified and contrasted between wild and selected lines to understand the early evolutionary consequences of captivity and selective breeding. To test for parallel domestication effects, genomic scans were performed on bulked wild versus selected sample sets to focus the outlier analysis on genetic changes shared by most selected strains and therefore more likely associated with inadvertent selection from standard‐practice culture conditions. Our results contribute to a growing literature on the speed and impacts of domestication effects by documenting genomic responses to selection shared among existing oyster strains and reporting multiple metrics of genomic change that accompanied differentiation from wild populations.

## 2 MATERIALS AND METHODS

### 2.1 Wild and selected strain oyster samples

**FIGURE 1:**
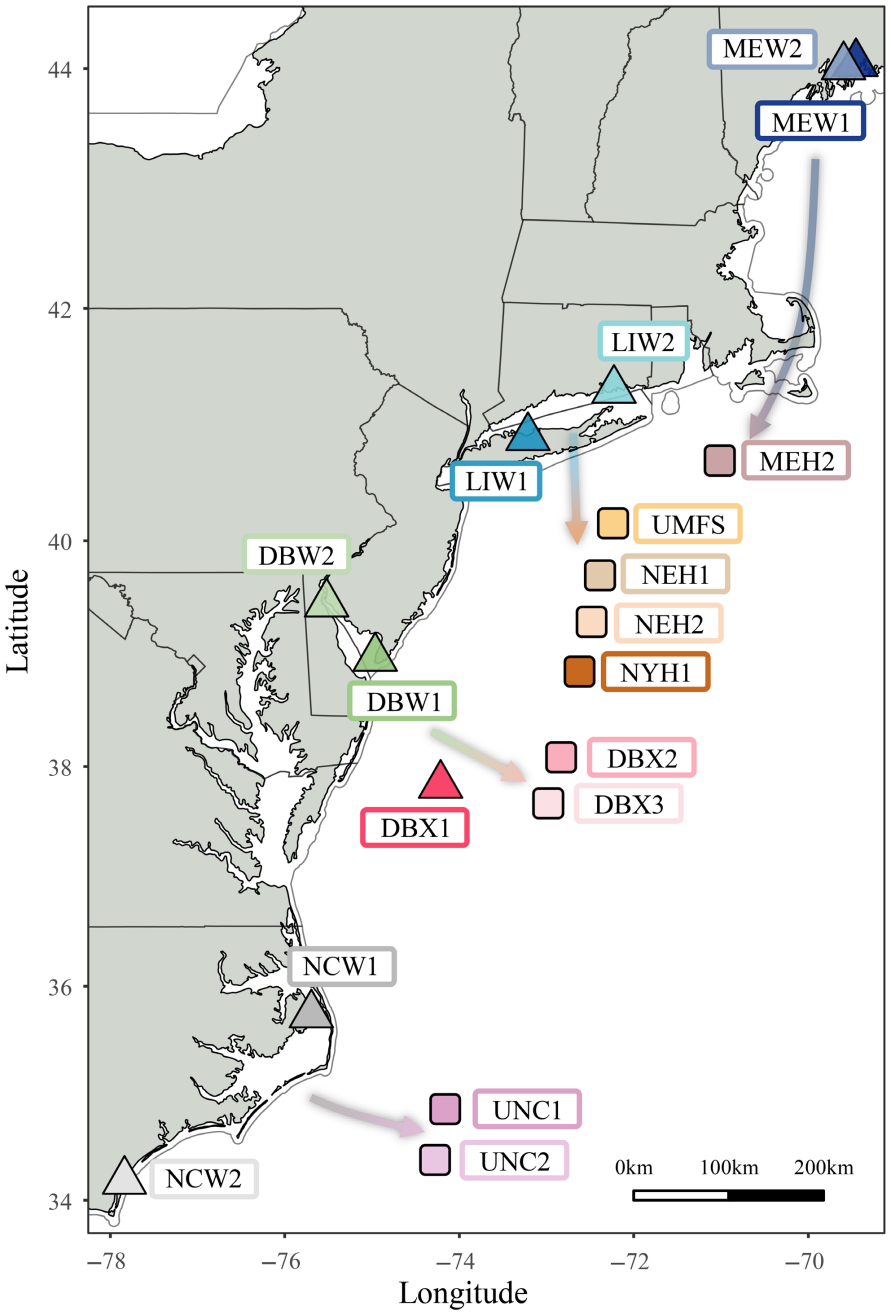
Map of wild oyster samples and approximate geographic source (arrow) for selection lines studied. Wild oyster population samples are marked with triangles, while selected lines are squares.

A total of 960 oyster samples were genotyped with a 566K bi‐allelic single nucleotide polymorphism (SNP) Affymetrix Axiom array (Guo et al., 2023). Among them, adult eastern oysters from nine wild populations and nine selected lines (1–17 generations of breeding) were selected for the current domestication study (Figure 1), with ~32 samples per site (but see Table S1 for exceptions). The wild oyster populations were collected in the fall of 2020 from four Atlantic locations: a pair of adjacent rivers in Maine, two estuaries in the Mid‐Atlantic (Long Island Sound [LIS] and Delaware Bay [DB]), and two estuaries in North Carolina. At each regional location, wild populations inhabiting two different sites were sampled. “Wild” is used here to designate naturally recruited oysters, not to imply that they are necessarily pristine or undisturbed. The selected lines include MEH2 from a Maine hatchery stock, ME; UMFS originally sourced from LIS stock and subsequently selected for fast growth and resistance to ROD and MSX in the Damariscotta River, ME; NEH1 and 2 selected for disease resistance in Delaware Bay but originated from Long Island Sound, NY; NYH1 as a hatchery line derived from Long Island Sound for ROD resistance since 1992; a pair of DBX derivative lines from Delaware Bay sourced lines founded in 1960 (DBX2 and 3); UNC1 and 2 that were sourced from wild North Carolina oysters in 2012 and selected for fast growth. One sample falls between these “wild” and “selected strain” designations; DBX1 is the F1 cohort produced by crossing wild samples from Delaware Bay. DBX1 is labeled “wild” in Results because it was not subject to selective breeding, but it experienced one generation of potential inadvertent selection in culture. Detailed population information, abbreviations, GPS coordinates, selection history, and sample sizes are listed in Table S1.

More specifically, NEH1 was a mixture of 4 NEH sublines. One of the sublines contains a small amount of genetic material from the Gulf of Mexico (~5%), but the majority ancestry is LIS. NEH2 (21 females × 39 males) is 100% LIS and one of the pure NEH sublines. Several NEH sublines were maintained with different levels of inbreeding, and then crossed in rotation for the production of hybrid vigor (Guo, 2021). The earliest DBX line was produced in 1960 from DB wild oysters. Multiple DBX derivative lines were developed over the years by crossing DBX sublines with NEH, NEG, or occasionally with DB wild individuals. NEG designates NEH male × Louisiana female hybrids backcrossed to NEH. DBX1 is the F1 offspring of wild DB individuals (18 females × 12 males) collected in June 2016 and sampled after 4 years of outplant at Cape Shore, New Jersey. Standard Rutgers hatchery practice included minimal loss of the smallest larvae during sieving for culture maintenance and settlement of the early (largest) pediveligers. Typically, survival from D‐stage to pediveligers is 25%–45% (McDonald et al., 2023), setting success of pediveligers to spat is ~40%–60%, and from spat to 4‐year‐old at Cape Shore is 15%–45% or 38% for DBX1 (Guo Lab, unpublished). DBX2 (35 females × 25 males) was produced in 2020 from a DBX line that was crossed with NEH. DBX3 (33 females × 26 males) is a hybrid stock whose parentage included NEH × DBX F1 hybrid, DB wild, and NEG. The selected line UNC1 is derived from 2012 broodstock collections from the same reef as NCW1 (Crab Hole, NC). The UNC2 line is derived from oysters collected in 2012 from the same reef as NCW2 (Hewletts Creek, NC). Both UNC1 and UNC2 went through two generations of selection along closed lines. Microsatellite pedigree analysis indicates that the UNC1 sample resulted from 5 crosses (10 individuals) and the UNC2 oysters resulted from 19 pair crosses (38 individuals).

### 2.2 SNP genotyping and filtering

DNA extraction and genotyping with the eastern oyster 566 K SNP array (CvSNP600) were described by Guo et al. (2023). Axiom Analysis Suite 4.0 (Thermo Fisher Scientific, MA, USA) was used to process the raw array data and call SNPs with QC thresholds (dQC ≥0.82, call rate ≥ 97%) recommended by the Best Practices Workflow. Genotypes of “BestAndRecommended” SNPs were exported into Variant Call Format (VCF). A total of 299,899 BestAndRecommended SNPs were used as the starting point for the following filtering steps (Guo et al., 2023). Mitochondrial SNPs on the array were excluded. SNPs located in putative inversions (Puritz et al., 2022, 2023) were excluded to prevent artifacts in population structure patterns from long‐range linkage disequilibrium. Additional filtering steps were applied using VCFtools (Danecek et al., 2011): excluded SNPs with minor allele frequency < 0.05 across 18 wild and domestic populations combined; individuals were removed if they had >10% missing data; SNPs with a call rate less than 95% in any single population were excluded. SNPs departing from Hardy–Weinberg equilibrium (HWE) with a *p*‐value <0.01 in at least 50% of the populations were removed using the script filter_hwe_by_pop.pl from the dDocent pipeline (Puritz et al., 2014). The quality filtering described above produced a dataset hereafter referred to as “full SNPs.”

To further minimize artifacts in population structure analyses from linkage disequilibrium (LD) and long‐range LD (for example, inversions), we applied “clumping” in a 10 kb sliding window keeping pairs of SNPs with *r* ^2^ < 0.2 using the function snp_autoSVD as implemented in the bigsnpr package (Privé et al., 2018). This function consists of the following steps: first, for SNP pairs with higher correlation, the SNP with a higher minor allele frequency is retained (i.e., LD clumping), and then principal component analysis (PCA) is performed using a subset of SNPs remaining after LD clumping. Finally, it utilizes an iterative algorithm that automatically detects and removes SNP outliers with heavy weights (loadings) in the PCA. The resulting SNP set after snp_autoSVD is referred to as “clumped SNPs” hereafter. The SNP count summary during each filtering step is presented in Table S2. The names of SNP subsets, the number of SNPs, and their usage are listed in Table S3.

### 2.3 Genomic scan for convergent response to domestication selection

To identify loci where genetic drift can be rejected as a mechanism causing high differentiation, potentially identifying regions showing a response to differential selection between selected stocks and their regional source population, we utilized two different genome scan methods: OutFLANK v.0.2 (Whitlock & Lotterhos, 2015) and PCAdapt v.4.1.0 (Luu et al., 2017). Outlier detection using OutFLANK was conducted using the “best practice” approach, in which neutral parameterization was done with the clumped SNPs and testing was performed on the full SNPs (Lotterhos, 2019). We implemented OutFLANK with a default expected heterozygosity cutoff (Hmin = 0.1) and a *q*‐value cutoff of 0.05. Similarly, we utilized the clumped SNP for neutral parameterization and performed PCAdapt outlier detection on the full SNPs. We used multiple values of K (1–10) to capture the major principal components summarizing variation in the data. Single SNP outliers were identified with a *q*‐value threshold of 0.05. After outlier detection, we created a “clumped neutral SNPs” dataset by excluding all PCAdapt and OutFLANK outliers from the “full SNPset,” followed by LD clumping as described above.

Using the union of SNPs identified with these two methods (hereafter referred to as combined outliers), we performed random forest (RF) analysis to identify the most informative SNPs for individual assignments, distinguishing wild from selected oysters. Random forest (RF) analysis was performed with the R package randomForest (Liaw & Wiener, 2002). To optimize the number of trees (ntree), we looped over a parameter space from 1 to 1000 trees to calculate the out‐of‐bag error rate (OOB‐ER). The OOB‐ER is an estimate of the misclassification of out‐of‐bag samples when using the classification model (Brieuc et al., 2018). Meanwhile, the number of predictors (mtry) considered for each node was evaluated using different mtry settings: 34, 68, 117, 234, 391, and 1174, corresponding to the square root of p, two times the square root of p, 10% of p, 20% of p, one‐third of p, and p, where p is the number of combined outliers (*n* = 1174, see Section 3) for RF running (Brieuc et al., 2018). We set the training data as 2/3 of all samples (randomly drawn with replacement) and the remaining 1/3 of samples as test data. The sample size parameter was used in conjunction with strata to ensure an equal representation of all strata in unbalanced experimental data (Brieuc et al., 2018).

For marker selection, we followed a two‐step backward purging approach to develop a group of SNPs that best discriminate between wild and selected strain oysters (Brieuc et al., 2015). Briefly, the first round of RF running was performed using combined outliers to estimate the importance value for each SNP. The performance (measured using OOB‐ER) of forests from various subsets of the most important loci (e.g., top 1%, top 5%, and top 10%,) was estimated in order to determine a candidate group of loci that minimizes the OOB‐ER. Second, backward purging was then conducted on a conservative candidate list of loci (e.g., using the top 5% of loci if the top 4% SNPs appeared to have the lowest OOB‐ER) to account for any uncertainty in the importance ranking of the loci. During this step, a single SNP with the lowest average importance value (averaged across three replicates) was removed, and the process was repeated iteratively until two SNPs remained for RF running. The SNP set with the lowest OOB‐ER, referred to as “RF outliers,” was deemed to be predictive of phenotype (i.e., wild vs. selected in this case). We explored the cluster pattern of individuals inferred from RF outliers using a discriminant analysis of principal components (DAPC) function implemented in the R package Adegenet (Jombart & Ahmed, 2011).

We extracted the gene annotation for combined outliers from the eastern oyster genome v3.0 (https://www.ncbi.nlm.nih.gov/assembly/GCF_002022765.2/). GO terms were assigned to all quality filtered “full SNPs” using EggNOG mapper v 2.1.4 (Cantalapiedra et al., 2021). We then performed gene ontology (GO) enrichment analyses using R package clusterProfiler v4.0.5 (Wu et al., 2021). Enrichment tests were used to identify statistically overrepresented GO terms among the combined outliers contained within genes and also in the subset of RF outliers, relative to the universe of annotations represented by the “full SNPs” dataset, with a default hypergeometric statistical test and a false discovery rate of 0.1.

### 2.4 Population structure, genetic diversity, relatedness, and effective population size

Population structure analyses were performed with the clumped neutral SNP dataset. Principal component analysis (PCA) was utilized to visualize population structure in the clumped neutral SNPs using the snpgdsPCA function implemented in SNPRelate 3.15 (Zheng et al., 2012). The Bayesian clustering algorithm implemented in STRUCTURE version 2.3.4 (Pritchard et al., 2000) was used to characterize population admixture patterns using the clumped neutral SNPs. The admixture model with correlated allele frequencies was applied with a burn‐in of 10,000 iterations followed by 100,000 Markov chain Monte Carlo (MCMC) repetitions. We used different numbers of assumed population genetic clusters (K = 1–18 for the whole dataset with 18 populations, and K = 1–10 for subsets of samples) to determine the best‐supported K values using the program Kfinder (Wang, 2019), repeated 10 times for each K using EasyParallel (Zhao et al., 2020). Three criteria are implemented in Kfinder to evaluate the support for each K value: Pr[X|K] (Pritchard et al., 2000), ΔK (Evanno et al., 2005), and parsimony index (Wang, 2019). These are all reasonable heuristic criteria and yet they often disagree, so we present all supported results.

The clumped neutral SNPs were used to calculate population diversity indices for each population, including observed (*H*_o_) and expected (*H*_e_) heterozygosity and allelic richness (A_r_) using hierfstat v 0.5‐10 (Goudet, 2005). Relatedness was estimated for each oyster population using the R package Demerelate v 0.9‐3 (Kraemer & Gerlach, 2017). The Ritland estimator was used because it has been shown to have the least bias with small sample sizes (Ritland, 1996). Population differentiation was estimated for all pairs of populations using the unbiased Weir & Cockerham estimator of *F*_ST_ (Weir & Cockerham, 1984) implemented in hierfstat.

Contemporary effective population size (*N*_e_) for each population was estimated from clumped neutral SNPs using the single‐sample linkage disequilibrium method (Hill & Weir, 1988; Waples & Do, 2010) and a random‐mating model implemented in NeEstimator version 2.1 (Do et al., 2014). Estimates of linkage disequilibrium (LD) from a mixed‐age sample (i.e., wild) provide an estimate of the effective population size (*N*_e_) per generation at the time of sampling, whereas using the same procedure on a single cohort (all aquaculture strain samples) estimates the effective number of breeders (*N*_b_) that produced the sample. Both estimators provide information about the risks of inbreeding and genetic drift (Waples & Antao, 2014). Random 5K SNPs were used for the computational efficiency of *N*_e_ estimation, with alleles <0.05 frequency excluded. The option of “LD locus pairing across chromosomes” was turned on to exclude SNP comparisons within a chromosome because they can have elevated LD from physical linkage (Waples et al., 2016). Confidence intervals were estimated based on the jackknife method implemented in NeEstimator.

It was previously hypothesized that the relative spread of genotypes in PCA plots (ellipse size) is associated with population admixture levels (Barrett & Schluter, 2008). Given that population admixture levels and relatedness both vary within this dataset (see Section 3), we tested these effects on standardized PCA ellipse size using a two‐way ANOVA. We used the *stat_ellipse* function in ggplot2 to draw 95% confidence‐level ellipses. The area of each PCA ellipse was approximated by measuring the semi‐major (A) and semi‐minor axes (B) and calculating their product with pie, πAB. The mean admixture level was measured by the Shannon‐Wiener index using the STRUCTURE admixture coefficient as input, where the admixture coefficient is obtained from STRUCTURE assignment tests using K = 6 (see Figure S1 for clustering results). Population relatedness estimates were described above.

### 2.5 Linkage disequilibrium decay patterns

Linkage disequilibrium (LD) is the nonrandom association of alleles of different loci, and patterns of LD decay can provide insight into the population subdivision, bottleneck, admixture, and selection history (Slatkin, 2008). The LD for each SNP pair was estimated using PopLDdecay v. 3.41 (Zhang et al., 2019). The full SNP set was used for the LD decay analyses. To ensure that LD decay results are comparable among populations, we randomly sampled 30 individuals per population for examinations, with the exception of UNC populations (*n* = 20). The maximum distance for pairwise estimates was set at 500 kbp. Pairwise LDs were then binned into a range of pairwise SNP distances in 100 increments, and the average *r* ^2^ within each window was measured. This process was repeated for each of the 10 chromosomes in each of the 18 populations. The rate of LD decay was then fit with the Hill and Weir formula under a drift‐recombination equilibrium for finite sample sizes and a low level of mutation (Hill & Weir, 1988). The distance at which LD is half of its maximum value (i.e., half‐decay distance) was estimated for each chromosome and population. A two‐way ANOVA was used to assess the effect of population type and chromosomes and their interaction on the half‐decay distance.

### 2.6 Runs of homozygosity

Runs of homozygosity (ROH) represent long tracks of homozygous genotypes and are formed when descendants inherit identical by descent DNA from a common ancestor. Measurement of ROH can provide insight into the population inbreeding history, with a larger population having fewer and shorter ROH while populations experiencing small population size or a recent bottleneck will have more and longer ROH (Ceballos et al., 2018). Measurement of ROH is often preferable to other inbreeding estimators because of its precise and unbiased features (Kardos et al., 2018), but also because the genomic distribution of ROH can be informative about variation in recombination rates. Runs of homozygosity (ROH) were investigated following a standardized protocol (Gorssen et al., 2021) using Plink v 1.9 (Chang et al., 2015). Full SNPs were used as input for ROH analysis. None of the loci were filtered based on minor allele frequency (‐‐maf), Hardy–Weinberg equilibrium test (‐‐hwe), or LD thinning, as evidence shows that MAF filtering and LD thinning can be problematic for ROH detection with medium density array data (Meyermans et al., 2020). The ROH analysis was conducted by population, in which each individual must have a genotype call rate >90% (‐‐mind 0.10), and samples with relatedness higher than 95% (‐‐genome; PI_HAT >0.95) were removed. The minimal SNP call rate was set to 0.95 (‐‐geno 0.05), and only populations with more than 15 individuals were retained after quality control. For ROH analysis, only one heterozygous SNP was allowed within a run (‐‐homozyg‐window‐het and –homozyg‐het), and five SNPs were allowed to be missing (‐‐homozyg‐window‐missing). The minimum number of SNPs per window (L, or ‐homozyg‐window‐snp in Plink) and per ROH segment (same as L, ‐‐homozyg‐snp) were population‐specific and calculated using the formula below (Purfield et al., 2012), where *n*_*s*_ is the number of genotyped SNPs per individual, *n*_*i*_ the number of genotyped individuals, *α* the percentage of false‐positive ROH (0.05), and $\overset{―}{\mathrm{het}}$ the mean heterozygosity across all SNPs.

$$L=\frac{\ln\frac{\alpha }{n_{s}n_{i}}}{\ln\left(1-\overset{―}{\mathrm{het}}\right)}$$

The scanning window threshold (*t*, or –homozyg‐window‐threshold) is estimated as follows,

$$t=\text{floor}\left(\frac{N_{\text{out}}+1}{L},3\right)$$

where *N*_out_ is the desired number of final outer SNPs on either side of the homozygous segment and *L* is the scanning window size (in SNPs). After ROH segment calling, extra parameters were used for segment filtering: a minimum segment length of 200 kb (‐‐homozyg‐kb), at least 1 SNP every 50 kb (‐‐homozyg‐density), and a maximal interval of 1000 kb between two SNPs in a segment (‐‐homozyg‐gap). The identified ROH was categorized based on different length cutoffs: 0.2–0.5, 0.5–1, 1–2, 2–4, and 4–8 Mb.

Genomic inbreeding was also measured according to McQuillan et al. (2008) and defined as:

$$\;F_{\mathrm{ROH}}=\frac{\Sigma L_{\mathrm{ROH}}}{L_{\text{TOTAL}}}$$

where Σ*L*_ROH_ is the sum of ROH (SROH) across the individual genome, and *L*_TOTAL_ *is* the total size of the autosomal genome covered by SNP array data. Here, *L*_TOTAL_ is estimated by simulating an individual with a completely homozygous genotype (using Plink .map file) and performing ROH analysis with the same specified parameters. Therefore, the *L*_TOTAL_ of this simulated individual represents the maximal detectable summed ROH length (Meyermans et al., 2020). Genomic *F*_ROH_ values between wild and selected groups were compared using a Kruskal–Wallis test.

## 3 RESULTS

### 3.1 SNP filtering and density

Filtering out putative inversions removed 96.7 Mb, and haplotig‐masked regions removed 100.4 Mb from consideration, leaving 276,327 SNPs. After strict quality filtering, the SNP array dataset (“full SNPs”) consisted of 141,676 SNPs and 539 individuals (Table S2). The average density was one SNP per 3.4 kb in the full SNP dataset (Table S3). The clumped SNPs with lower LD consisted of 106,456 markers and one SNP per 4.6 kb. SNP counts during each filtering step are presented in Table S2.

### 3.2 Outlier loci from selected‐wild population contrast

**FIGURE 2:**
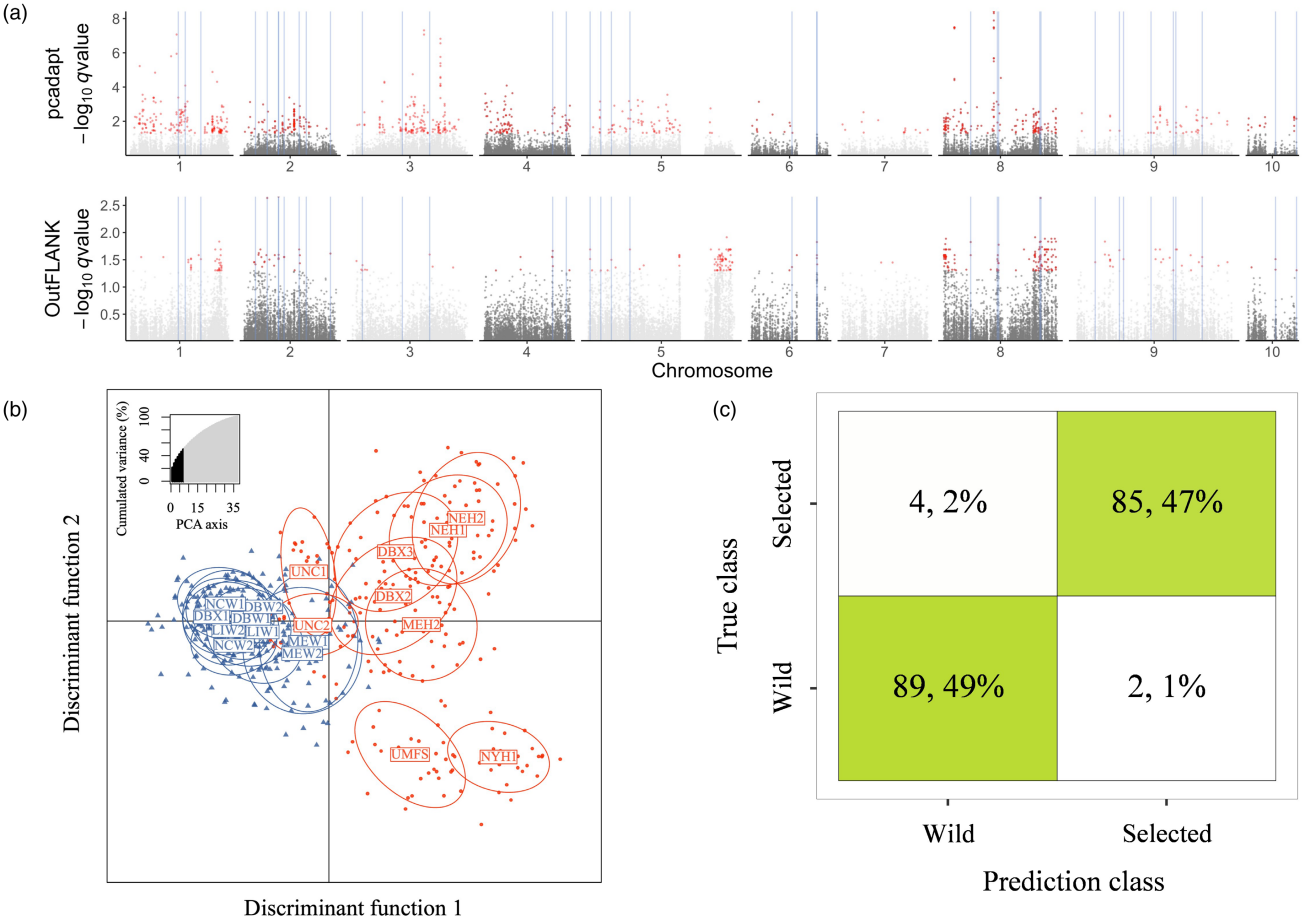
Outlier candidate loci from wild versus selected oyster contrasts. (a) Manhattan plots showing 1174 outlier loci identified with two independent genome scan methods (red) and random forest (RF) identification of the most differentiated SNP loci highlighted with blue vertical lines. (b) Genetic clusters and 95% confidence ellipses inferred from DAPC using 37 Random Forest (RF) outlier SNPs. (c) Confusion matrix obtained from measuring classification accuracy on RF outliers. Green boxes highlight the prediction that matches the actual labels, while white boxes show the mismatch between the prediction and the actual labels.

We reasoned that artificial selection on each strain is likely to have generated distinct genomic responses, whereas domestication selection pressures, that is, selection from the culture environment, produce a common signal of differentiation relative to progenitor wild populations. Thus, population samples were combined into two groups, with individuals labeled as wild or selected for outlier detection using genome scans of the full SNP dataset. Using a *q*‐value <0.05 cutoff, PCAdapt identified 918 and Outflank identified 327 outlier SNP candidates (Figure 2a). After deduplication (71 of 1245 SNPs are shared), the 1174 union outlier SNPs were tested for enrichment of GO terms. Among them, 656 out of 1174 SNPs fall within the annotated genes. Twelve enriched terms were identified with FDR < 0.1, and only one GO term (GO:0008307 for structural constituent of muscle) was enriched at FDR < 0.05 (Table S4).

To identify the smallest subset of markers that can accurately distinguish wild versus selected strains of oysters, we performed random forest analysis using the 1174 union outlier SNPs. Because out‐of‐bag error (OOB‐ER) stabilized at approximately 1000 trees (ntree) and 391 predictors (mtry) during the parameter optimization step (Figure S2), we accepted these as suitable settings for the rest of the RF analyses. The RF importance values across SNP candidates indicated that the top 4% of SNPs minimized the OOB‐ER (Figure S3). A two‐step backward purging approach identified 37 random forest outliers (i.e., RF outliers). DAPC analysis with RF outliers could distinguish between wild and selected individuals (Figure 2b). The confusion matrix obtained from measuring classification accuracy on RF outliers showed high assignment accuracy (96%), with most mismatches coming from MEW and UNC populations that were intermediate in the PCA (Figure 2c). No significantly enriched GO terms were identified among the 37 RF outliers.

### 3.3 Population structure

**FIGURE 3:**
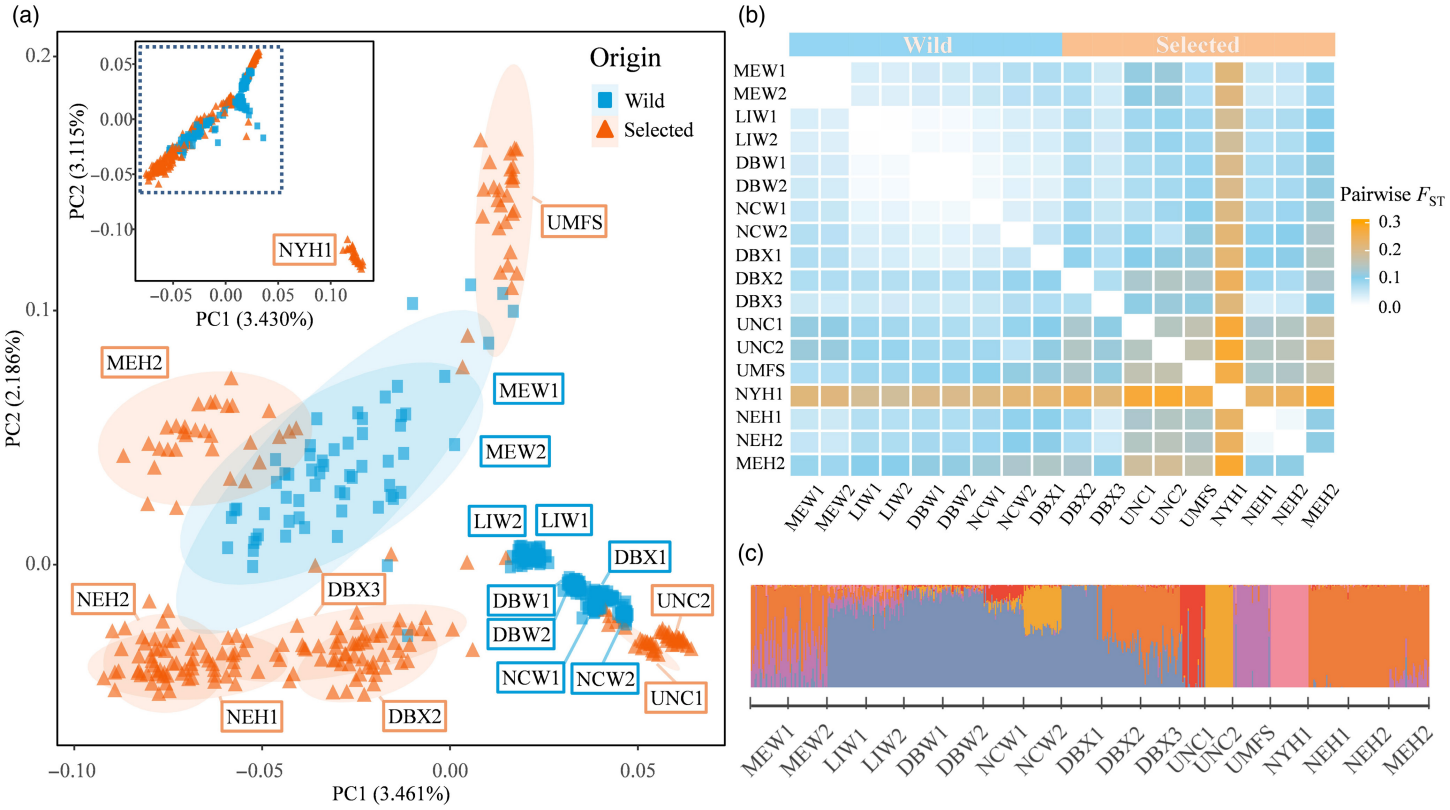
Population structure and genetic differentiation among examined oyster populations. (a) Population structure inferred with principal component analysis using principal components (PC) axes 1 and 2. (b) Heat map of pairwise population genetic differentiation based on *F*_ST_. Locality abbreviations are explained in Table 1. (c) STRUCTURE clustering results using clumped neutral SNPs with K = 6.

To make inferences about evolutionary demography across populations with diverse selection histories, we chose to exclude candidate SNPs and focus analysis on presumably neutral genomic variation by using the clumped neutral SNPs dataset. Principal component analysis (PCA) identified a relatively large genetic differentiation between the selected line NYH1 and all other populations (Figure 3a inset). Excluding NYH1 for an expanded view of the other populations along pricipal component (PC) axes 1 and 2, wild populations are mostly clustered in the center and lower‐right versus selected strains in the margins around wild clusters (Figure 3a). Both wild populations from Maine are intermixed with selected strains and show a broader dispersion of genotypes relative to the other, tightly clustered wild populations. In contrast, UNC1 and UNC2 are unique among the selected strains by clustering in the lower right with wild populations, and they show tight population clusters similar to the wild population pattern (Figure 2b). Selected NEH and DBX populations are more dispersed and well separated from their wild populations. The PC1 and PC2 axes explained similar proportions of variance, 2.186%–3.461%.

Genome‐wide average *F*_ST_ in pairwise contrasts ranged from −0.0010 to 0.2798 when based on all SNPs (Figure 3b). The *F*_ST_ values among selected line contrasts had the highest average (mean = 0.1490, range 0.0138–0.2798), contrasts between wild populations had the lowest average (0.0314, −0.0010 to 0.0683), and wild population contrasts with selected lines were intermediate (0.0936, 0.0405–0.2237). In contrast, the 1174 union outlier SNPs generated mean *F*_ST_ values of 0.3815, 0.1865, and 0.3272, respectively.

Inferring the number of differentiated populations, K, based on assignment tests using an admixture model provided different results from Pr[X|K], ΔK, and Parsimony Index criteria (Figure S1). These heuristic criteria can provide useful guidance but are known to often disagree and be inaccurate when applied to complex datasets (Wang, 2019). In this case, ΔK identified K = 3, where the UMFS strain was not resolved as distinct from the wild. The other two criteria highlighted K = 9 and 10 as the most probable number of source populations, but these each show unexplainable admixture within the simple F1 DBX1 cohort (wild × wild). The K = 6 result is the most parsimonious and useful (Figure 3c) because differences are recognized between NCW1 and NCW2 (in agreement with Varney et al., 2016), UNC1 and UNC2 are distinguished from wild, and DBX2 has admixture from NEH and wild in agreement with its breeding history, rather than being depicted as an unadmixed unique strain (Figure S1). At K = 6, there is no geographic population structure inferred among wild populations that is not more easily explained as admixture from selected strains (Figure 3c). Certain wild oyster populations consistently showed admixture with selected lines across multiple K, including K = 6, particularly within Maine (MEW1 and MEW2) but also in Long Island Sound, New York (LIW), and North Carolina (NCW). Selected lines were mostly distinct from each other except for MEH2 and two sublines (DBX2, DBX3) of the oldest selected strain, NEH. The DBX2 and DBX3 strains have complex breeding histories, but at K = 6 or less, they share a nearly even admixture pattern combining NEH and wild (Figure S1). The DBX1 line has the shortest history of captivity, with only a single generation of hatchery propagation of Delaware Bay wild individuals, consistent with a structure profile with fully wild allele frequencies (Figure 3c).

### 3.4 Genetic diversity and effective population size

Mean observed heterozygosity (*H*_o_), expected heterozygosity (*H*_e_), allelic richness (A_r_), relatedness, and effective population size (*N*_e_) were calculated for each population using the clumped neutral SNPs (Table 1). *H*_o_ was similar between the wild and selected lines, with an average of 0.242 and 0.236, respectively (Wilcoxon rank‐sum test, *p*‐value = 0.6019), whereas *H*_e_ in the wild (mean 0.272) was significantly greater than that for the selected lines (mean 0.243; Wilcoxon rank‐sum test, *p*‐value = 0.0020). All but MEH2, UNC1, and NYH1 populations displayed higher levels of *H*_e_ than *H*_o_, that is, a deficit of observed heterozygous sites relative to Hardy–Weinberg expectations. Allelic richness was significantly larger in the wild populations (mean 1.852) compared to selected lines (mean 1.717, Wilcoxon rank‐sum test, *p*‐value = 0.0002).

**TABLE 1:** Diversity indices and effective population size (*N*_*e*_) statistics for each *Crassostrea virginica* sampling site, including allelic richness (A_r_), observed heterozygosity (*H*_o_), expected heterozygosity (*H*_e_), relatedness and its standard deviation, effective population size or number of breeders (*N*_*e*_ or *N*_b_, after excluding minor allele frequencies <0.05) and their 95% confidence intervals (CI).

Group	Sites	*N*	A_r_	*H*_o_	*H*_e_	Relatedness	*N*_e_ or *N*_b_ (CI)
Wild	MEW1	30	1.836	0.241	0.271	0.091 (0.070)	189.3 (119.3, 429.9)
	MEW2	31	1.845	0.241	0.272	0.085 (0.062)	360.1 (241.4, 692.7)
	LIW1	31	1.865	0.246	0.271	0.057 (0.061)	158.9 (44.7, ∞)
	LIW2	30	1.867	0.248	0.272	0.057 (0.038)	10,412.8 (1484.3, ∞)
	DBW1	31	1.870	0.242	0.275	0.058 (0.034)	43,677.6 (1897.5, ∞)
	DBW2	32	1.873	0.241	0.277	0.058 (0.037)	19,722.8 (3236.1, ∞)
	NCW1	32	1.870	0.240	0.277	0.089 (0.044)	12,916.2 (2163.3, ∞)
	NCW2	30	1.842	0.232	0.267	0.131 (0.043)	21,493.1 (2667.9, ∞)
	DBX1	32	1.803	0.243	0.263	0.147 (0.148)	15.9 (12.8, 20)
Mean		31	1.852	0.242	0.272	0.086	12,105.2
Selected	DBX2	31	1.767	0.239	0.257	0.200 (0.104)	52.3 (36.8, 83.7)
	DBX3	31	1.843	0.251	0.276	0.136 (0.109)	42.3 (32, 59.4)
	UNC1	20	1.722	0.247	0.245	0.290 (0.254)	2.4 (1.6, 4.7)
	UNC2	22	1.702	0.226	0.238	0.318 (0.119)	24.9 (15.3, 50.4)
	UMFS	30	1.711	0.231	0.239	0.311 (0.165)	37.5 (19.5, 124.3)
	NYH1	30	1.454	0.185	0.171	0.610 (0.085)	64.4 (54.3, 78.6)
	NEH1	32	1.810	0.246	0.267	0.176 (0.088)	127.3 (92, 200.5)
	NEH2	32	1.781	0.247	0.260	0.204 (0.095)	57.3 (38.5, 100.2)
	MEH2	32	1.665	0.250	0.234	0.334 (0.159)	12.1 (8.8, 16.5)
Mean		29	1.717	0.236	0.243	0.287	46.7

The relatedness measured within selected lines was significantly larger than in the wild populations (Table 1; Wilcoxon rank‐sum test, *p*‐value = 8.2e‐05). The NYH1 population displayed the highest level of relatedness among selected strains (max = 0.610, average 0.287), while wild LIW1 and LIW2 showed the lowest level of relatedness (min = 0.057, average 0.086). Estimates of *N*_e_ in wild populations with no hatchery propagation ranged from 158.9 to 43677.6 (average = 13616.4, excludes DBX1). The DBX1 hatchery cohort was an outlier with *N*_b_ = 15.9, more similar to the selected line range of *N*_b_ from 2.4 (UNC1) to 127.3 (NEH1, Table 1; average = 46.7). All wild population estimates were larger than selected line estimates, except for the DBX1 population. Wild populations showed infinite values at upper jackknife confidence limits, except for the highly admixed Maine populations.

To explore factors contributing to variation in PCA ellipse size (Figure 1a), a two‐way ANOVA was performed on standardized PCA ellipse size with mean admixture and relatedness as explanatory factors (Table S5). Simple main effects analysis showed that only mean admixture had a strong effect on standardized ellipse size (*F* = 9.721, *p* = 0.008). No interaction effect was found between mean admixture and relatedness in a separate test, *F* (1, 14) = 0.055, *p* = 0.818.

### 3.5 Linkage disequilibrium decay within populations

**FIGURE 4:**
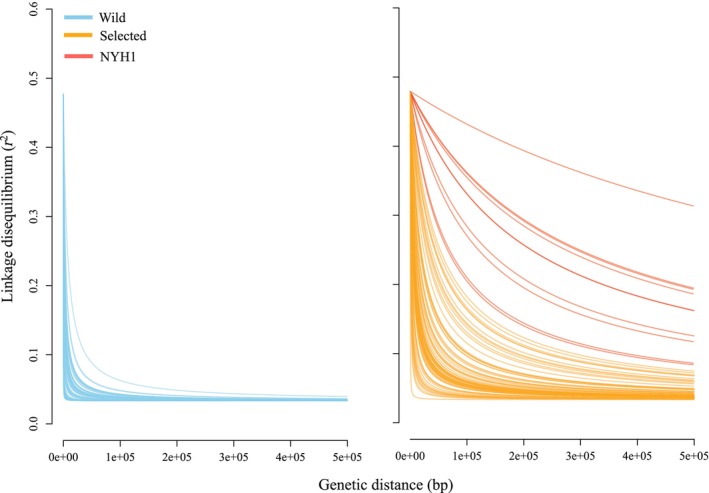
Linkage‐disequilibrium decay patterns for all separate chromosomes in selected lines and wild oyster populations. Wild oyster populations (blue) show narrower LD decay than selected lines, even after accounting for the NYH1 selected line that has an especially high LD.

Linkage disequilibrium (LD) decayed faster with distance along chromosomes in wild populations than in selected lines. The half‐decay distance was significantly greater in selected populations than in wild populations (Wilcoxon rank‐sum test, *p*‐value < 0.001), with a median value of 7250 bp versus 450 bp, respectively (Figure 4). Based on ANOVA, there was a significant relationship between half‐decay distance and chromosome identity (*df* = 9, *F* = 54.003, *p*‐value < 0.001), the population sources (wild vs. selected, *df* = 1, *F* = 38.244, *p*‐value = 1.311e‐05), as well as the interaction between these two factors (*df* = 9, *F* = 12.546, *p*‐value = 1.433e‐14).

### 3.6 Runs of homozygosity

**FIGURE 5:**
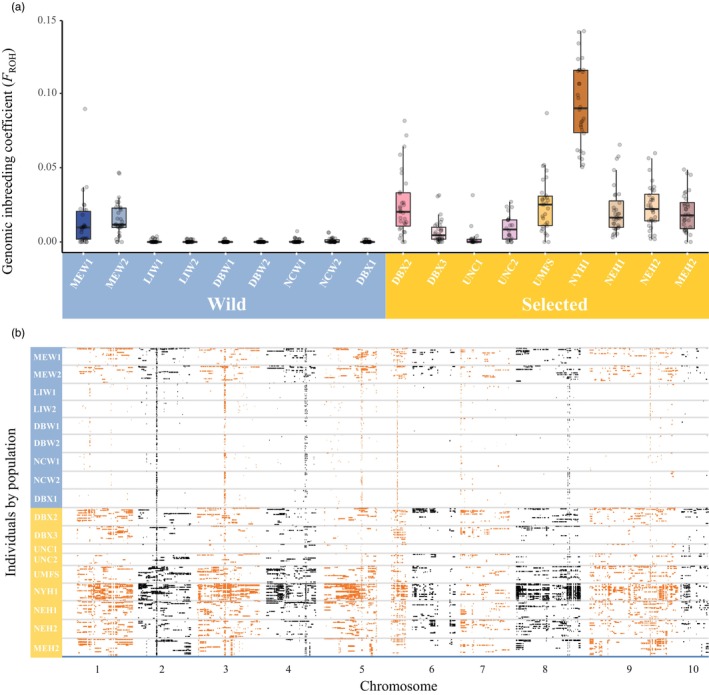
Comparison of genomic inbreeding (*F*_ROH_) and ROH distribution between selected lines and wild populations. (a) Genomic inbreeding estimated from ROH, with inbreeding calculation made for ROH >1 Mb. (b) Distribution of ROH along chromosomes on the *X*‐axis for individuals in each selected and wild population.

The genome inbreeding coefficient based on runs of homozygosity (*F*_ROH_) is a superior and accurate metric for individual inbreeding as it does not rely on allele frequencies or sampling procedures (Ceballos et al., 2018; Talebi et al., 2020). ROH was initially analyzed by populations, in which every individual must have a call rate among SNPs >90%. A total of 534 out of 539 individuals were retained after this QC step. Shorter ROH are more prone to false positives, so to avoid this and maximize comparability between wild and selected strain populations, we considered ROH segments >1 Mb in length. Excluding short ROH has the added benefit of minimizing the background relatedness caused by ancestral bottlenecks (Foote et al., 2021). An order of magnitude larger proportion of the genome in selected lines was identical by descent (*F*_ROH_ mean = 0.0253) compared to wild populations (mean = 0.0035) (Kruskal–Wallis test: *χ* ^2^ = 226.59, *df* = 1, *p* < 0.001, Figure 5a). MEW1 and MEW2 displayed *F*_ROH_ higher than other wild populations and comparable to most selected strains (Figure 5a; Table S6), while NYH1 had the highest level of inbreeding observed in the selected populations. Visualization of ROH segments along chromosomes (Figure 5b) highlights the striking contrast of nearly zero ROH (>1 Mb) in most wild populations versus more extensive ROH in selected lines. In addition, narrow genomic regions show uniform enrichment for ROH in both wild and selected populations (ROH islands or hotspots).

## 4 DISCUSSION

This study documents genomic changes associated with the early phase of domestication in eastern oysters. Rapid genetic differentiation documented among selected strains and between them and progenitor wild populations was potentially caused by isolation, genetic drift, directed and undirected artificial selection, and/or inbreeding in captivity. Based on a genomic comparison between pooled wild oysters and pooled selected lines, substantial parallel genomic differentiation was apparent among selected strains after just a few generations of captive breeding, consistent with high evolutionary potential under directional selection. This rapid evolutionary response may be related to the life history characteristics shared by many marine species, including eastern oysters, that promote high genetic diversity from spatially balanced selection at many loci in the wild (Nunez et al., 2021).

Results on the oyster lines examined here indicate that inbreeding was typically kept at low levels (F < 0.05), maintaining conditions for effective selection. The average pairwise *F*_ST_ among all the wild versus selected strain contrasts was 0.094 for the full‐filtered SNPset. The especially high *F*_ST_ values at 1174 outlier SNPs (0.327 average, 0.059–0.772 min–max) for wild versus selected strains are indicative of parallel domestication effects in the studied breeding lines. Each strain represented 1/9th of the total selected strain pool, so even large‐effect selective sweeps, if unique to a strain, would not have generated a significant outlier. The conditions necessary for these outliers to rapidly become significantly differentiated above the drift background, convergently in multiple strains, include strong and similar selection pressures across the breeding lines, trait architectures that include large effect loci, and a moderate frequency of alleles at those loci in wild populations to promote a rapid per‐generation domestication response (Brennan et al., 2019; Charlesworth, 2006; Kemper et al., 2014). Because complex polygenic traits respond to selection with small allele frequency changes at many loci, as described by the infinitesimal model (Boyle et al., 2017; Fisher, 1918), the large number of *F*_ST_ outliers detected here may be better explained by parallel selection on multiple oligogenic traits. However, this study was not designed to infer the genetic architecture of domestication in bivalves, only to test whether genomic changes are rapid and parallel.

We expect that some of the selection signals identified here may be false positives experiencing genetic drift, and ultimately, the robustness of our parallel evolution inference will need to be tested in additional oyster strains not included here, especially those with fully independent breeding histories. Nonetheless, rapid increases in yield are generally reported to result from artificial selection on bivalves (Langdon et al., 2003), and faster growth is ubiquitously selected to improve commercial value (Guo, 2021; McDonald et al., 2023). In all hatcheries, pediveligers are given a limited amount of time to set, and any larvae that would not set quickly are eliminated. In addition to directed selection on juvenile and adult growth rates, an unknown number of traits may be inadvertently selected in culture. In fact, standard practice for keeping larval cultures healthy is to periodically drain tanks and catch larvae on a sieve that lets the smallest larvae pass through, truncating the lower end of the size distribution. As typically practiced (Davis et al., 2021), this sieving is not expected to generate strong selection for faster larval growth, but it is an example of how selection forces in culture can interact with directed selection on commercially valuable traits after field deployment. Thus, the relative importance of domestication selection in culture versus artificial selection for commercially valuable traits cannot be determined in this study, but there are two factors that suggest domestication selection in culture may be the dominant process generating convergent genomic patterns of differentiation. First, larval and setting mortality together in hatcheries are typically much larger than after field deployment (McDonald et al., 2023). Second, even a ubiquitous commercial trait like fast growth, when selected in different environments, can lead to distinct genomic outcomes because genetic by environment interactions are prominent in this species (Hughes et al., 2017; Proestou et al., 2016). In contrast, domestication selection for each strain occurs under essentially the same hatchery conditions and practices.

### 4.1 Genetic diversity and population structure

The patterns of genetic diversity revealed a decreased level of genetic variation in domesticated oysters after breeding management. This is expected, as most of the domesticated populations will experience an inevitable loss of genetic diversity due to limited connectivity with other populations and selective breeding (Baumung et al., 2004). The results of this study are consistent with a previous analysis in the eastern oyster, where selected populations showed similar heterozygosity but significantly reduced allele richness compared with wild populations (Yu & Guo, 2006). Continual monitoring of selected lines is needed to ensure that genetic diversity is not seriously eroded.

Coupled with the reduced genetic diversity, a higher level of genetic differentiation was observed among the selected lines than was observed among the wild populations. These results are in agreement with previous work that compared the genetic diversity (heterozygosity) and differentiation (*F*_ST_) among wild Atlantic salmon and domesticated strains after approximately 10 generations of domestication/captive breeding (Mäkinen et al., 2015). A similar observation was found when contrasting wild/domestic population pairs of Atlantic salmon from Canada and Scotland (López et al., 2019) or Pacific oyster population pairs throughout the Northern Hemisphere (Sutherland et al., 2020). Interestingly, only small deviations in heterozygosity from the Hardy–Weinberg expectation were reported in the salmon studies, an indication of heterozygosity excess due to the recent reduction in effective population size (Cornuet & Luikart, 1996; López et al., 2019; Mäkinen et al., 2015). In this study, only three selected populations (UNC2, MEH2, and NYH1) showed heterozygosity excess, while the other populations displayed a deficit of heterozygosity relative to Hardy–Weinberg. This finding is consistent with heterozygote deficiencies that were previously reported in oysters and other bivalves, with multiple factors (e.g., presence of null alleles, nonrandom sampling, selection, inbreeding, and population subdivision) potentially leading to the observed patterns (Appleyard & Ward, 2006; Borsa et al., 1991; English et al., 2000; Fairbrother & Beaumont, 1993; Gaffney et al., 1990; Guo et al., 2023; Lapegue et al., 2014; Thongda et al., 2018; Vercaemer et al., 2006; Waples & Antao, 2014). Sweepstakes reproductive success in shellfish is another factor potentially leading to population subdivision (i.e., Wahlund effect) and, therefore, deficiencies in heterozygosity (Bernatchez et al., 2019; Hedgecock & Pudovkin, 2011). Analysis of SNP array data from eastern oyster families has shown that a significant proportion (13.1%) of loci have null alleles (Guo et al., 2023), which may account for some of the heterozygote deficiency observed here.

The population structure revealed by the PCA and STRUCTURE analyses indicated there are distinct genetic patterns between wild and selected populations. The greater dispersion of selected strain individuals in PCA is likely driven by admixture, as hybridization is known to increase genetic variability (Barrett & Schluter, 2008). Indeed, we quantified the association between standardized ellipse size and mean admixture among sampled populations and found a significant positive effect. Admixture was characteristic of selected strains, presumably reflecting attempts to lessen inbreeding by crossing lines or introducing wild broodstock. However, two wild populations from Maine also showed evidence of hybridization or population introgression. With Structure K = 6, the two Maine wild populations showed significant components from MEH2, UMFS, and/or NEH, three of the selected stocks that have been used for oyster farming in Maine. Thus, these Maine samples appear to be feral selected strains that have become admixed, perhaps explaining why they also showed (historical) inbreeding levels higher than every other wild population. The current data are not sufficient to determine whether the Maine samples contained some remnant endemic genetic variation.

### 4.2 Effective population size and inbreeding profiles

Previous estimates of *N*_e_ in wild oyster populations based on microsatellite markers have ranged from 37 to 437 in Delaware Bay (He et al., 2012), 535 to 1516 in the James River (Rose et al., 2006), 75 to 130 in the Choptank tributary of the Chesapeake (Hornick & Plough, 2019), and using SNPs, Hornick and Plough (2022) estimated 124 to 501 in Chesapeake samples. The majority of estimates in this study, the first to be based on medium‐density SNP data, were an order of magnitude larger than any previous estimates, despite similar population sample sizes, but still suffered from infinite upper confidence limits, as is commonly reported. Hornick and Plough (2022) presented *N*_e_ estimates from re‐analyzed Canadian eastern oyster reduced representation SNPs, from data published by Bernatchez et al. (2019), and the *N*_e_ range (237–7071) was more similar to our findings here. Several mechanisms could potentially generate a downward bias on single sample LD‐based estimates of *N*_e_ (Waples et al., 2016; Waples & Antao, 2014). Given the infinite upper confidence limits on estimates here, we interpret the *N*_e_ results estimated in this study to indicate that wild eastern oyster populations remaining in many regions are large enough to be at low risk from genetic drift and inbreeding (Frankham et al., 2014). The especially small LD‐based *N*_e_ estimates in Maine wild samples presumably are biased downward by their admixture history. For the Long Island populations, the small *N*_e_ estimate for LIW1 (159) and its contrast with LIW2 (10,413) have no simple explanation based on diversity and admixture results.

Inbreeding and reduced effective population size are major concerns in selective breeding programs (Appleyard & Ward, 2006). In the current study, two measures of effective size, both measuring so‐called inbreeding effective size of the immediate or recent parental generations (Hare et al., 2011), were necessary because selected strain samples consisted of a single age‐class cohort, whereas mixed‐age adults were sampled from wild populations. Specifically, the number of breeding individuals estimated to have produced a hatchery cohort (*N*_b_) was much smaller compared to the effective population size per generation (*N*_e_) estimated in wild progenitor populations. For iteroparous species with different life histories and overlapping generations, *N*_b_ can be lower or higher than *N*_e_, but comparative data indicate *N*_b_ < *N*_e_ in many invertebrates (Waples et al., 2013). For a particular species and life table, the ratio of *N*_b_ to *N*_e_ depends mostly on adult lifespan, age of first reproduction, and variance in fecundity with age, although high variance in reproductive contributions may shift the relative importance of these factors (Waples et al., 2013). However, in captivity, the limited number of broodstock will typically lower *N*_b_ relative to *N*_b_ in wild‐sampled single‐age cohorts (Hornick & Plough, 2022). Also, here we have not accounted for admixture LD potentially created within selection lines from breeding practices designed to counter inbreeding (e.g., progressive rotational crossbreeding; Guo, 2021). Using LD to estimate *N*_b_ in admixed selected strains potentially generates a downward bias because admixture LD gets misinterpreted as inbreeding LD. In contrast, selection seems to have minimal effects on LD‐based estimates of *N*_e_ (Novo et al., 2022). For some of the selected lines, the *N*_b_ estimates were close to the actual numbers of parents used: DBX2, 52 vs. 60; DBX3, 42 vs. 59; and NEH2, 57 vs. 60. The relatively high average *N*_b_ = 46.72 observed among the selected strains (Table 1; see also Hornick & Plough, 2019) reflects optimized broodstock pairing designs that minimize sperm competition (Guo, 2021). In other cases, like the DBX1 hatchery cohort, in which neither admixture nor artificial selection were strong factors, its small *N*_b_ = 15.9 (relative to the expected *N*_b_ of 28.8 from 18 females x 12 males) reflects the potential for strong effects from variance in reproductive success in addition to broodstock number and sex ratio. In this case, the sample was taken 4 years after outplanting at Cape Shore, after 62% mortality had occurred, perhaps reflecting natural selection acting to increase variance in reproductive success within the cohort.

Small broodstock numbers for captive propagation are often associated with higher levels of relatedness among cohort individuals, in addition to elevated inbreeding (Waples & Do, 2010). This is well supported here by the three‐fold higher mean relatedness in selected strains compared to wild population samples. The highest relatedness was detected in the NYH1 selected strain sample, consistent with its high estimated inbreeding (*F*_ROH_); the relatively large *N*_b_ for this sample suggests that many broodstock are being spawned, but the whole breeding population is inbred.

The DBX1 cohort is of interest because it represents a single generation of hatchery propagation from wild parents, identical to what typically gets used for oyster restoration. In the hatchery, eastern oyster larvae typically experience 25%–45% survival from D‐stage to pediveligers that are competent to settle (McDonald et al., 2023), only half of which may successfully set as spat. When harvesting DBX1 larvae for setting, pediveligers were selected by size, a common practice, selecting for fast growth or fast development to reach settlement competence. After hatchery setting in June 2016, DBX1 spat were deployed in low intertidal racks at Cape Shore, NJ, in September 2016, then sampled in October 2020 for SNP array genotyping. Environmental conditions (disease and stress) over 4 years led to 62% mortality, perhaps selective. After this single generation of hatchery propagation and selection, DBX1 showed very slight decreases in allelic richness and heterozygosity relative to field‐collected adult Delaware Bay wild samples (Table 1). The DBX1 cohort had lower relatedness than all selected strains, and it did not show any evidence of inbreeding relative to the Delaware Bay wild samples. One individual had a 50/50 admixture pattern similar to the DBX2 and DBX3 and may have been a contaminant, or one of its parents in the hatchery cross was not truly wild. The lack of DBX1 differentiation from wild (for K = 6) contrasts with results for UNC1 and UNC2, each differentiated from North Carolina wild, even though the UNC strains experienced only 2 generations of selection. There may be differences in the strength of the selection that was imposed.

Inbreeding is an important genetic metric that helps to evaluate the fitness of a population, and monitoring inbreeding is critical for sustainable selective breeding. We relied on genome‐wide measurements of ROH for an absolute estimate of inbreeding magnitude. The distribution of ROH can also reveal the genomic regions that are responsible for inbreeding depression (D'ambrosio et al., 2019). As predicted, the *F*_ROH_ values indicated significantly stronger inbreeding in selected lines than in wild populations. However, the degree of inbreeding among selected populations was relatively small (*F*_ROH_ ranges from 0.003 in UNC1 to 0.0925 in NYH1, mean = 0.0253) compared to other aquatic species under selection, like farmed coho salmon (0.004–0.152, mean 0.099) (Yoshida et al., 2020) or commercial rainbow trout lines (0.100–0.195, mean 0.140) (D'ambrosio et al., 2019). The overall low level of *F*_ROH_ in the NEH and DBX strains studied here may result from the maintenance and crossing of multiple sublines during breeding (Guo, 2021), a protocol that is commonly utilized to minimize the negative impact of inbreeding depression (Zenger et al., 2019). A similar observation has been reported in coho salmon, where a recently admixed line with careful *N*_e_ and inbreeding controls showed a significantly lower level of *F*_ROH_ compared to other farmed salmon populations (0.004 in the control group vs. 0.152 or 0.142 in uncontrolled farmed populations) (Yoshida et al., 2020). Nonetheless, it is recognized that a *F*_ROH_ comparison across species is difficult to interpret due to different chromosome architectures and recombination rates (Curik et al., 2014).

Measurement of ROH can provide insight into the population history and demography over time, with a larger population harboring fewer and shorter ROH compared with a small or bottlenecked population having more and longer ROH (Ceballos et al., 2018). The ROH distribution of selected strains UNC1 and UNC2 is similar to that of other wild populations. For UNC1 and UNC2, this likely reflects a short breeding history with only two rounds of captive breeding. We also visualized ROH segments along the genome, aiming to find genomic regions linked to selection (i.e., ROH islands shared by selected strains) or with low recombination. Almost every chromosome had one ROH island, each equally uniform and prominent across wild and selected individuals. We hypothesize that these represent low recombination centromeric regions within *C*. *virginica* chromosomes, which are mostly metacentric or submetacentric (Wang et al., 2005; Xu et al., 2001). Note that chromosome 5 has two ROH islands, but chromosome 6 has none, likely caused by a known misassembly involving these two chromosomes (Puritz et al., 2022, 2023).

### 4.3 Genome scan for domestication signals

One aspect of domestication is the inadvertent but unavoidable selection to culture conditions, potentially involving many traits. With eastern oysters, similar hatchery practices among hatcheries might make this source of selection relatively uniform compared with artificial selection for commercially valuable traits. Thus, we hypothesized parallel domestication selection pressures across independent breeding lines. The admixture results show that not all selected lines are fully independent, but the elevated *F*_ST_ among selected strains indicates that gene flow was too limited to prevent differentiation by genetic drift given the small *N*_e_ in selected strains. Thus, it is plausible that these semi‐independent lines had the potential to also diverge by selection, and phenotypic breeding accomplishments support the role of directed artificial selection. For example, in a review by Gjedrem and Rye (2018), oysters were reported to experience a 10.3% gain in average body weight per generation of selection. With respect to domestication selection, the degree to which gene flow confounds a parallel selection mechanism remains ambiguous with the pooled contrast made here. In the future, individual strain by progenitor wild contrasts might indicate relative strengths of domestication selection at the loci identified here if pairwise estimation of gene flow between strains was also used to identify portions of the genome more or less subject to inter‐strain gene flow.

The distinction made here between inadvertent domestication traits and directed commercial traits is not new (Zohary, 2004). Our informal knowledge of eastern oyster hatchery practices is what supports the conjecture that hatchery culture selection pressures are uniform across strains relative to field‐based artificial selection for commercially valuable traits. Logically, if candidate loci identified here were affected by parallel selection across breeding lines (i.e., true positives), then pooling oyster strains will have strengthened power for their detection. In contrast, pooling is expected to obscure any genomic responses to selection that were unique to single strains, and because genetic drift also creates unique changes in isolated lines, both mechanisms of differentiation would be unlikely to produce significant outliers in a pool of selected strains. One commercial trait may have enough ubiquity among eastern oyster breeding programs that it could produce parallel selection patterns—growth rate. Increasing growth rates is a common goal among breeders of eastern oyster strains along the Atlantic U.S. coast (Allen et al., 2021; Davis & Barber, 1999; Guo, 2021; Rawson & Feindel, 2012). With the study design used here, we are unable to separate generalized domestication effects from the impact of selection for faster growth, either applied during larval culture or setting or applied after field deployment.

Our prediction was that signals associated with recent domestication might be hard to detect due to short‐term captive breeding and complex quantitative trait architecture (Mäkinen et al., 2015).

We performed two different genome scan approaches and used a machine‐learning framework to localize the strongest genomic responses to captive selection. We observed a genomically pervasive distribution of outliers, indicating a polygenic architecture of domestication traits when considered all together. Polygenic traits are controlled by multiple loci with small or moderate effects, and “outliers” are not necessarily expected or detectable (Gagnaire & Gaggiotti, 2016). With large sample sizes, moderate effect loci might be detectable as outliers, but architecture redundancy (many allele combinations can make the same trait value) could make the identity of outliers depend on starting conditions (genetic background; Bourret et al., 2014). Here, the lack of congruence in outlier candidates detected by PCAdapt and OutFLANK was likely due to the different statistics underlying each approach (López et al., 2019). Their distinct approaches motivated functional enrichment testing on the 1174 SNP union results, and 7 molecular function and 5 related biological process GO terms occurred among the candidates more often than expected (FDR <0.1). The 78 genes annotated with these GO terms almost all relate to cytoskeletal or muscle proteins and protein kinases (Table S4), suggesting that culture tank environments and perhaps selection for fast growth may be selecting for developmental or structural changes to the cytoskeleton or adductor muscle (Epelboin et al., 2016). The myosin heavy chain, striated muscle‐like gene (LOC 111120907) is represented in many of these GO enrichment categories (Table S4) and was identified as a marker gene for a cluster of anterior mesoderm cells expressing myogenic genes in trochophore larvae of *Dreissena rostriformis* (Salamanca‐Díaz et al., 2022). Staining of filamentous actin has also been used to map dynamic myogenesis at multiple larval stages of *Crassostrea gigas*, followed by degeneration of muscle structures at metamorphosis and development of mantle, gill, and posterior adductor musculature in spat (Li et al., 2019). Also, all selected oysters have a free‐living lifestyle, while wild oysters are attached to substrate, which may impact muscle function. Further work will be needed to identify which myogenic processes and structures are responding to domestication. Given that ~50% mortality is common at the hatchery larval settlement stage (selection can be strong), and settlement success is a trait that was shown to differ significantly between one selected strain and its wild progenitor population (McDonald et al., 2023), settlement and metamorphosis may be an important life history stage where domestication selection has early effects.

These functional genetic inferences are tentative because the high LD in oyster selected strains that we and others have documented (Xuereb et al., 2023) makes it unlikely that identified outliers are the direct target of selection. During a few generations of positive selection on small populations in early domestication, there is little opportunity for meiotic recombination to separate selection effects on target SNPs from surrounding polymorphisms. As a result, linked selection can create broad chromosomal footprints with the appearance of polygenic architecture, even when the architecture is oligogenic. Further work, including other bivalve species, will be needed to confirm the relevance of these GO terms and these candidate genes for bivalve domestication and link them to traits experiencing selection in the hatchery or after field deployment. Because gene expression patterns sometimes mirror functional evolutionary responses to selection, it may be informative to compare gene expression patterns in fast versus slow growing larval fractions, or at high versus low larval density to test for culture conditions that trigger differential expression of these candidates at various early developmental stages. For example, dynein motor proteins were identified as one of the gene types whose expression correlated with early larval shell deposition and showed sensitivity to CO_2_‐acidified seawater or perhaps water quality more generally (De Wit et al., 2018).

We also used a random forest method to identify the outlier union SNPs with the greatest information content to discriminate selected strains from wild samples. Random Forest is a suitable approach to search for signals of polygenic selection by accounting for the correlation and interactions among loci (Boulesteix et al., 2015). It has been previously applied for polygenic locus detection between control and polluted ecotypes in North Atlantic eels (Laporte et al., 2016) or polygenic signatures related to run timing in Chinook salmon (Brieuc et al., 2015). Our analysis based on only the 37 RF outlier SNPs was successful at separating wild and selected strain oysters with 96% accuracy, indicative of the high information content of these loci. These RF outlier variants are potentially valuable assets for biosecurity management and for investigating the consequences of selective breeding.

We acknowledge that ascertainment bias in the construction of this high‐density SNP array, as with most SNP arrays designed for purposes other than population genetics, generates some risk of false positive outliers (Nielsen, 2004). Some of the specific SNPs identified as highly informative using RF on these array data may reflect ascertainment biases, so future applications should start with a larger set of outliers identified here in addition to the 37 RF SNPs. The comparison of genetic differentiation across all SNPs as opposed to the 1174 union candidate SNPs is only partly consistent with our domestication interpretation for outlier loci. The outlier loci generate a mean pairwise *F*_ST_ among selected strains (0.3815) that is 2× higher than that among wild populations (0.1865), showing a trend in the expected direction. However, the mean *F*_ST_ for domestication outliers among wild populations is 5.9× higher than the 0.0314 mean estimated with all SNPs, whereas true domestication SNPs arguably are not expected to have above‐average differentiation among wild populations. Among the selected strains, the outlier loci generate an average *F*_ST_ that is 2.6× the value based on all SNPs. This suggests that loci identified here as domestication candidates may be confounded with loci that have high differentiation among wild oyster populations.

There are specific contexts where the RF SNP candidates, or the outliers in general, may be less diagnostic of selected and wild status. For example, RF misassignments exist in populations with short‐term breeding management (DBX1 and UNC1, two genetic assignment mismatches) or groups from Maine inferred to be feral (MEW1 and MEW2, three mismatches). Although assignment power was lower in eastern oyster populations at very early stages of domestication, performance here was better than predicted by Mäkinen et al. (2015), who found the need for >10 generations of domestication selection based on simulating domestication selection on quantitative trait evolution. Our results also suggest that individuals with complex admixture backgrounds may be less accurately assigned, consistent with the observation that Random Forest self‐assignment accuracy was relatively low in admixed Atlantic salmon populations (Sylvester et al., 2018).

## 5 CONCLUSION

In addition to the relatively strong genetic drift expected in small breeding lines, early domestication and artificial selection in the eastern oyster have led to pronounced genomic changes in selected strains compared to native progenitor populations. Contrasting pools of selected oyster populations versus their approximate wild source stocks revealed significant differences in standing genetic diversity, inbreeding, and runs of homozygosity profiles, as well as genome‐wide linkage disequilibrium patterns. Genetic drift has accompanied domestication in patterns consistent with breeding history, and breeding management to minimize inbreeding appears to be effective in many cases. Parallel genomic signatures of domestication selection were only expected in the multiple strains studied if oyster culture conditions or artificial selection imposed similar pressures across largely independent breeding programs. Indeed, the 1174 domestication SNP candidates were spread across the genome, and many occur in genes enriched for molecular functions that might be associated with changes in muscle development or cytoskeletal functions. Thus, these are candidate traits possibly selected differentially under culture relative to adaptations of free‐living early oyster life stages. This work highlights the effectiveness of eastern oyster breeding programs and provides genomic resources that can help investigate domestication consequences and efficiently distinguish wild from selected strain oysters for improved biosecurity management.

## FUNDING INFORMATION

The study is supported by a grant from the National Oceanic and Atmospheric Administration (NOAA) and the United States Department of Commerce through the Atlantic States Marine Fisheries Commission (award number NA18NMF4720321). XG and ZW are also supported by USDA NIFA Animal Health projects 1021665/NJ30401. The statements, opinions, findings, conclusions, and recommendations are those of the authors and do not necessarily reflect the views of NOAA, Department of Commerce.

## CONFLICT OF INTEREST STATEMENT

None declared.

## Supporting information


Appendix S1.

## Data Availability

Raw VCF from SNP array data and code used in this study are available at: https://github.com/hzz0024/Cv_domestication_SNP_array.
