# Supplementary material for: Consequences of domestication in eastern oyster: Insights from whole genomic analyses
Source: Evol Appl. 2024 May 29;17(6):e13710. doi: 10.1111/eva.13710 (PMC11134191; doi:10.1111/eva.13710)
Supplement: Supplementary file 1 — Appendix S1. [file EVA-17-e13710-s001.docx]

## **Supplementary Figures and Tables**


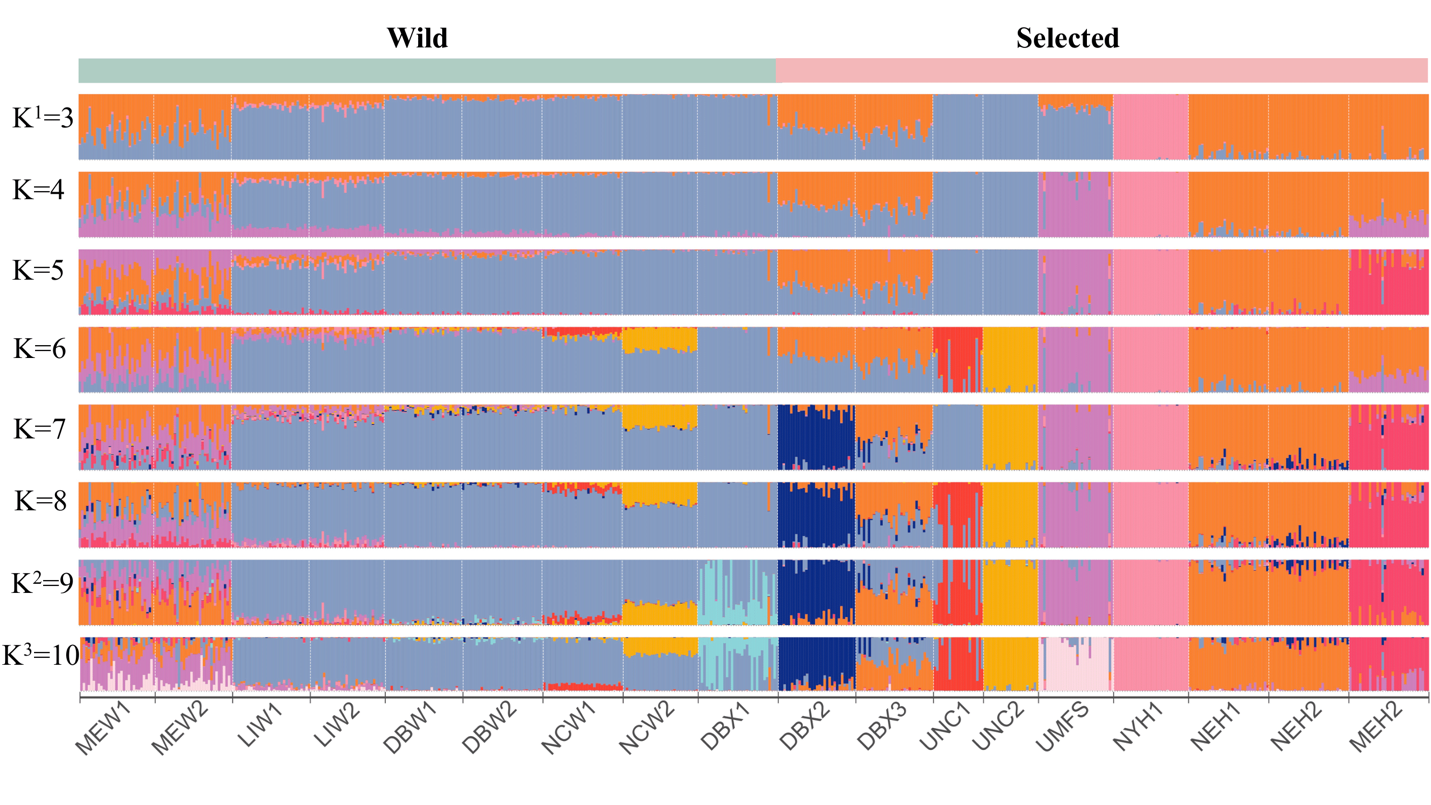
**Figure S1**. STRUCTURE assignment test clustering results using Clumped neutral SNPs for K= 3-10 with the most likely K values indicated using three criteria in KFinder: K^1^ – ΔK; K^2^ –parsimony index; K^3^ – Pr[X|K].

**Figure S2**. Convergence of the out-of-bag error rate (OOB-ER) across different number of predictor (mtry) values and trees (ntree) used in the random forest analysis. The out-of-bag error rate (OOB-ER) is optimized for ntree = 1000 and mtry = 391. The number mtry are set as 34, 68, 117, 234, 391, 1174 for sqrt(p), 2*sqrt(p), 0.1(p), 0.2(p), p/3, and p, where p is the number of total SNP used for analysis (1174 SNPs).


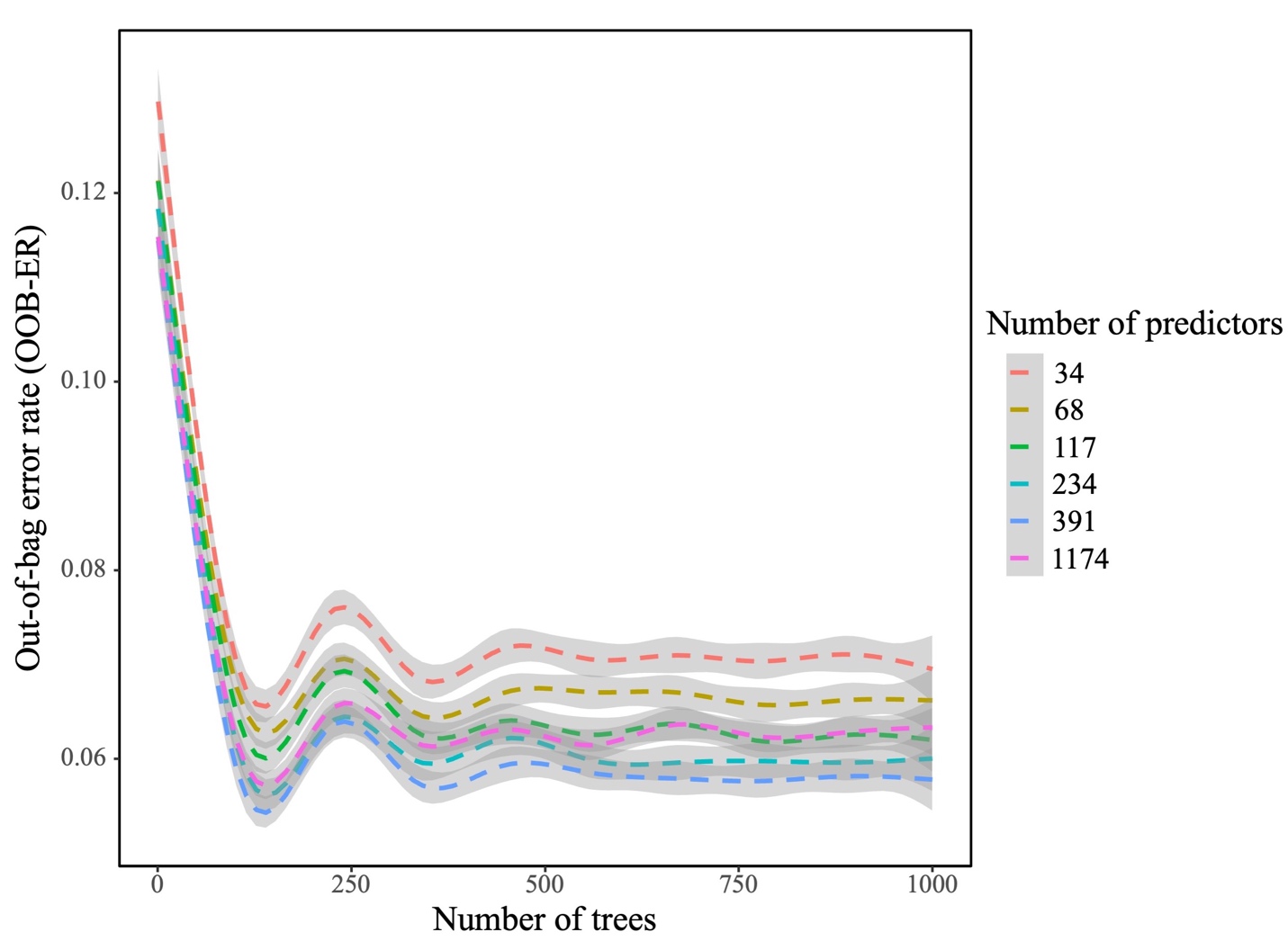


**Figure S3**. Two-step backward purging analysis for random forest outlier detection. (a) Performances of initial random forest analyses on all 1,174 loci and subsets of loci with the highest importance values. Out-of-bag error rate (OOB-ER) is an estimate of the misclassification of out-of-bag samples based on classification random forest model. Number of loci indicates that the top percentile loci minimized the OOB-ER, from left to right: top 2%, top 3%, top 4%, top 5%, top 10%, top 20%, top 30%, and all combined outlier 1,174 SNPs. (b) A second round of backward purging on the top 5% SNPs; the top 37 SNPs (red) maximized the OOB-ER and were considered RF outliers.


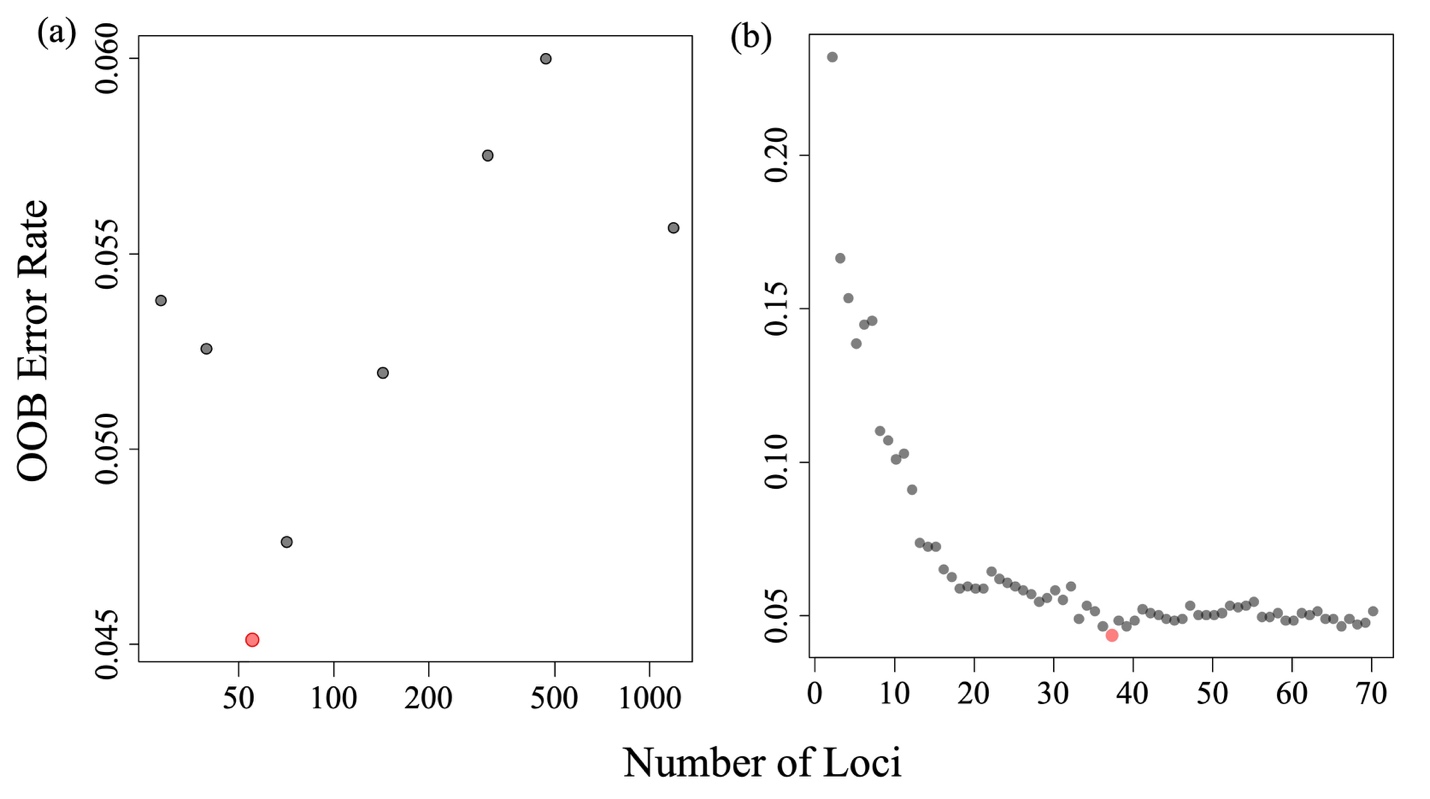


**Table S1**. Population information of eastern oysters used in domestication study. The population column lists the names of selected strains or the source locations of wild populations. Abbr indicates the short name of populations. N is the number of individuals in each population. Latitude and Longitude lists coordinates of the wild populations. Genetic source indicates the approximate ancestry composition in each population. F0 is the year domestication began and “Gen of Selection” gives approximate number of generations under selection and assumes that even a simple hatchery propagation involves some culture-based selection. Collection date is for this study.

| Population | Abbr | N | Latitude | Longitude | Genetic Source | F0 | | Gen of selection | Collection date |
| --- | --- | --- | --- | --- | --- | --- | --- | --- | --- |
| U. Maine UMFS | UMFS | 30 |  |  | 100% Long Island Sound | 1986 | | ~10 | 10/15/20 |
| Maine Hatchery Stock 2 | MEH2 | 32 |  |  | NA | NA | | NA | 9/22/20 |
| Rutgers NEH 19N1357 | NEH1 | 32 |  |  | ~95% Long Island Sound | 1966 | | 16 | 10/8/20 |
| Rutgers NEH 20N1 | NEH2 | 32 |  |  | 100% Long Island Sound | 1966 | | 16 | 10/8/20 |
| New York Hatchery Stock 1 | NYH1 | 30 |  |  | 100% Long Island Sound | 1966 | | 16 | 10/15/20 |
| Rutgers DBX WDB16 | DBX1 | 32 |  |  | 100% Delaware Bay | 2016 | | 1 | 10/8/20 |
| Rutgers DBX 20D1 | DBX2 | 31 |  |  | Delaware Bay and NEH | 1960 | | 17 | 10/8/20 |
| Rutgers DBX 20D4 | DBX3 | 31 |  |  | NEH, DBW, NEG hybrids | 1966 | | various | 10/8/20 |
| 2018 Crab Hole, UNC selected | UNC1 | 20 |  |  | 100% North Carolina wild | 2012 | | 2 | 10/2/20 |
| 2018 Hewletts Creek, UNC selected | UNC2 | 22 |  |  | 100% North Carolina wild | 2012 | | 2 | 10/2/20 |
| Hog Island, ME 1 | MEW1 | 30 | 44.013 | -69.541 | Wild caught |  |  | | 10/23/20 |
| Sheepscot, ME 2 | MEW2 | 31 | 44.014 | -69.595 | Wild caught |  |  | | 9/22/20 |
| Lloyd harbor, NY, Long Island Sound 1 | LIW1 | 31 | 40.896 | -73.212 | Wild caught |  |  | | 6/17/20 |
| Niantic Bay, CT, Long Island Sound 2 | LIW2 | 30 | 41.303 | -72.226 | Wild caught |  |  | | 7/7/20 |
| Cape Shore, Delaware Bay 1 | DBW1 | 31 | 38.968 | -74.962 | Wild caught |  |  | | 10/8/20 |
| Hope Creek, Delaware Bay 2 | DBW2 | 32 | 39.447 | -75.519 | Wild caught |  |  | | 10/19/20 |
| Crab Hole, North Carolina 1 | NCW1 | 32 | 35.728 | -75.675 | Wild caught |  |  | | 10/2/20 |
| Hewlett's Creek, North Carolina 2 | NCW2 | 30 | 34.179 | -77.841 | Wild caught |  |  | | 10/2/20 |
| Total |  | 539 |  |  |  |  |  | |  |

**Table S2.**  Summary of data filtering procedures: rows refer to filtering steps; columns refer to statistics for each step. For columns, ‘sites’ refers to SNPs, and ‘Inds’ refers to individuals. ‘Start’, ‘End’, and ‘Removed’ refer, respectively, to the number of each unit before the filtering step, the number after the filtering step, and the number removed with the filter.

| **Filter steps** | **Start sites** | **End sites** | **Start Inds** | **End Inds** | **Removed sites** | **Removed Inds** |
| --- | --- | --- | --- | --- | --- | --- |
| Exclude putative inversions, and loci in the mtDNA | 299,899 | 276,327 | 842 | 842 | 23,573 | 0 |
| Exclude irrelevant populations | 276,327 | 276,327 | 842 | 539 | 0 | 303 |
| Filter_missing_ind script; Ind call rate > 0.9 | 276,327 | 276,327 | 539 | 539 | 0 | 0 |
| Minor allele frequency > 0.05, genotype call rate > 0.95 | 276,327 | 147,160 | 539 | 539 | 129,167 | 0 |
| pop_missing_filter script; call rate > 0.95 in any single population | 147,160 | 147,160 | 539 | 539 | 0 | 0 |
| Hardy-Weinberg equilibrium | 147,160 | 141,676 | 539 | 539 | 5,484 | 0 |

**Table S3.** Name of SNP subsets, number of SNPs and their usage.

| SNP subset name | Analyses | Number of SNPs | Source |
| --- | --- | --- | --- |
| Full SNPs | Genetic scan (PCAdapt and OutFLANK); LD decay (PopLDdecay); runs of homozygosity (Plink) | 141,676 | SNPs after quality filtering |
| Clumped SNPs | Genetic scan (PCAdapt and OutFLANK) | 106,109 | Full SNPs after LD clumping |
| Combined outliers | Individual assignment (Random forest) | 1,174 | Union of PCAdapt and OutFLANK outliers (q < 0.05) |
| RF outliers | DAPC (Adegenet); RF assignment |  | Random forest (RF) Outliers identifed from combined outliers |
| Clumped neutral SNPs | Population structure (PCA and STRUCTURE); genetic diversity (Ho, He, Ar, and FST calculated by hierfstat); relatedness (Demerelate); | 105,672 | Excluding all PCAdapt and OutFLANK outliers in the full SNPs, followed by LD clumping (10K window size with r2 > 0.2) |
| Random 5K SNPs | NeEstimator | 5,000 | Excluding all PCAdapt and OutFLANK outliers in the full SNPs, followed by randomly selecting 5K markers |

**Table S4**. Summary of GO enrichment test results using 1174 union outlier SNP candidates. Ontology means categories from the GO database; MF - molecular function; BP - biological process; CC - cellular component. GO ID is identity number for the descriptive GO Term. GeneRatio indicates ratio between the number of genes annotated by a specific GO term among the outlier candidates and the total number of outlier SNPs that were within a gene with an annotation. BgRatio indicates the ratio between the number of genes annotated by a specific GO term across the full SNP set and the total number of annotated genes containing a SNP in this data set. FDR means false-discovery rate of GO significance. Gene ID is the *C. virginica* reference gene identity for the genes containing outlier SNPs having this GO annotation.

| Ontology | GO ID | GO Term | GeneRatio | BgRatio | FDR | Gene ID |
| --- | --- | --- | --- | --- | --- | --- |
| MF | GO:0008307 | structural constituent of muscle | 7/257 | 25/6661 | 0.019 | LOC111103558/LOC111108484/LOC111112221/LOC111118916/LOC111120907/LOC111124621/LOC111134715 |
| BP | GO:0030036 | actin cytoskeleton organization | 36/283 | 454/7424 | 0.066 | LOC111102987/LOC111104835/LOC111105753/LOC111105791/LOC111106754/LOC111107307/LOC111107462/LOC111108484/LOC111110125/LOC111112730/LOC111112846/LOC111114913/LOC111118072/LOC111118083/LOC111118411/LOC111118916/LOC111120143/LOC111120907/LOC111122663/LOC111122911/LOC111124621/LOC111125362/LOC111126608/LOC111127107/LOC111127258/LOC111128734/LOC111129057/LOC111129335/LOC111130255/LOC111130592/LOC111132799/LOC111133145/LOC111133318/LOC111134366/LOC111135851/LOC111138099 |
| BP | GO:0071688 | striated muscle myosin thick filament assembly | 5/283 | 11/7424 | 0.066 | LOC111108484/LOC111118916/LOC111120907/LOC111124621/LOC111135851 |
| MF | GO:0019901 | protein kinase binding | 34/257 | 470/6661 | 0.072 | LOC111099092/LOC111102894/LOC111103558/LOC111104835/LOC111105791/LOC111107462/LOC111107518/LOC111109993/LOC111110978/LOC111112730/LOC111113264/LOC111114913/LOC111115416/LOC111118004/LOC111118072/LOC111118083/LOC111118783/LOC111118916/LOC111120596/LOC111120676/LOC111122956/LOC111123971/LOC111124479/LOC111124927/LOC111125362/LOC111125791/LOC111125905/LOC111127107/LOC111129057/LOC111131917/LOC111132966/LOC111135944/LOC111136039/LOC111137241 |
| MF | GO:0004674 | protein serine/threonine kinase activity | 20/257 | 224/6661 | 0.072 | LOC111099092/LOC111106754/LOC111107462/LOC111108425/LOC111108484/LOC111109993/LOC111113264/LOC111118004/LOC111119812/LOC111120596/LOC111120676/LOC111124479/LOC111125119/LOC111125362/LOC111129057/LOC111129335/LOC111130075/LOC111130582/LOC111131917/LOC111137105 |
| MF | GO:0016773 | phosphotransferase activity, alcohol group as acceptor | 27/257 | 357/6661 | 0.072 | LOC111099092/LOC111105753/LOC111106648/LOC111106754/LOC111107462/LOC111108425/LOC111108484/LOC111109993/LOC111113264/LOC111115195/LOC111118004/LOC111118072/LOC111119812/LOC111120596/LOC111120676/LOC111124479/LOC111125119/LOC111125362/LOC111127107/LOC111127258/LOC111128435/LOC111129057/LOC111129335/LOC111130075/LOC111130582/LOC111131917/LOC111137105 |
| MF | GO:0000146 | microfilament motor activity | 4/257 | 11/6661 | 0.072 | LOC111118916/LOC111120907/LOC111124621/LOC111131555 |
| MF | GO:0003774 | cytoskeletal motor activity | 9/257 | 66/6661 | 0.081 | LOC111118916/LOC111119668/LOC111120585/LOC111120907/LOC111124621/LOC111125905/LOC111131555/LOC111134366/LOC111134888 |
| MF | GO:0004672 | protein kinase activity | 23/257 | 294/6661 | 0.081 | LOC111099092/LOC111105753/LOC111106754/LOC111107462/LOC111108425/LOC111108484/LOC111109993/LOC111113264/LOC111118004/LOC111119812/LOC111120596/LOC111120676/LOC111124479/LOC111125119/LOC111125362/LOC111127107/LOC111127258/LOC111129057/LOC111129335/LOC111130075/LOC111130582/LOC111131917/LOC111137105 |
| BP | GO:0031034 | myosin filament assembly | 5/283 | 13/7424 | 0.086 | LOC111108484/LOC111118916/LOC111120907/LOC111124621/LOC111135851 |
| BP | GO:0071539 | protein localization to centrosome | 6/283 | 21/7424 | 0.086 | LOC111099092/LOC111100168/LOC111107814/LOC111109832/LOC111126173/LOC111134366 |
| BP | GO:1905508 | protein localization to microtubule organizing center | 6/283 | 21/7424 | 0.086 | LOC111099092/LOC111100168/LOC111107814/LOC111109832/LOC111126173/LOC111134366 |

**Table S5.** Ellipse size, Structure admixture index, and relatedness among examined oyster populations. The area of PCA ellipse was approximated by measuring the semi-major (A) and semi-minor axes (B) and calculating their product with pie, πAB (Fig. 3a inset). The mean admixture level was measured by the Shannon-Wiener index using STRUCTURE admixture coefficient as input at K = 6 (see Fig. S1 for clustering results).

| Pop | Ellipse size | Admixture index | Relatedness |
| --- | --- | --- | --- |
| DBW1 | 1.18E-05 | 0.133 | 0.058 |
| DBW2 | 1.47E-05 | 0.049 | 0.058 |
| DBX1 | 4.18E-05 | 0.028 | 0.147 |
| DBX2 | 3.46E-04 | 0.677 | 0.200 |
| DBX3 | 8.11E-04 | 0.679 | 0.136 |
| LIW1 | 1.27E-04 | 0.515 | 0.057 |
| LIW2 | 1.78E-04 | 0.571 | 0.057 |
| MEH2 | 6.74E-04 | 0.591 | 0.334 |
| MEW1 | 1.13E-03 | 0.891 | 0.091 |
| MEW2 | 9.78E-04 | 0.904 | 0.085 |
| NCW1 | 1.37E-05 | 0.468 | 0.089 |
| NCW2 | 6.30E-06 | 0.646 | 0.131 |
| NEH1 | 6.73E-04 | 0.222 | 0.176 |
| NEH2 | 3.82E-04 | 0.074 | 0.204 |
| NYH1 | 2.37E-04 | 0.000 | 0.610 |
| UMFS | 1.62E-04 | 0.182 | 0.311 |
| UNC1 | 5.36E-05 | 0.314 | 0.290 |
| UNC2 | 3.13E-05 | 0.078 | 0.318 |

**Table S6.** Sum (in Kb) and number of ROH over individuals within each population. S_ROH_all_ means sum of ROH across all individuals within a population; SROH_1_2_ indicates sum of ROH from 1 to 2 Mb; SROH_2_4_ indicates sum of ROH from 2 to 4 Mb; SROH_4_8_ indicates sum of ROH from 4 to 8 Mb; SROH_>1_ indicates sum of ROH larger than 1 Mb; NSEG indicates number of ROH segments across individuals within a population; NSEG_1_2_ indicates number of ROH from 1 to 2 Mb; NSEG_2_4_ indicates number of ROH from 2 to 4 Mb; NSEG_4_8_ indicates number of ROH from 4 to 8 Mb; NSEG_>1_ indicates number of ROH larger than 1 Mb

| Pop | *F*_ROH_ | S_ROH_all_ | SROH_1_2_ | SROH_2_4_ | SROH_4_8_ | SROH_>1_ | NSEG | NSEG_1_2_ | NSEG_2_4_ | NSEG_4_8_ | NSEG_>1_ |
| --- | --- | --- | --- | --- | --- | --- | --- | --- | --- | --- | --- |
| MEW1 | 0.0131 | 75345.9 | 19264.4 | 5713.0 | 0.0 | 24977.5 | 1244 | 148 | 24 | 0 | 172 |
| MEW2 | 0.0155 | 78896.7 | 21774.2 | 8735.4 | 0.0 | 30509.5 | 1247 | 163 | 35 | 0 | 198 |
| LIW1 | 0.0006 | 8114.4 | 1086.0 | 0.0 | 0.0 | 1086.0 | 176 | 8 | 0 | 0 | 8 |
| LIW2 | 0.0004 | 8421.8 | 811.0 | 0.0 | 0.0 | 811.0 | 200 | 7 | 0 | 0 | 7 |
| DBW1 | 0.0002 | 5185.6 | 378.8 | 0.0 | 0.0 | 378.8 | 126 | 3 | 0 | 0 | 3 |
| DBW2 | 0.0002 | 5921.4 | 309.3 | 0.0 | 0.0 | 309.3 | 150 | 3 | 0 | 0 | 3 |
| NCW1 | 0.0007 | 7147.5 | 1432.8 | 0.0 | 0.0 | 1432.8 | 160 | 10 | 0 | 0 | 10 |
| NCW2 | 0.0009 | 8011.6 | 1194.3 | 506.7 | 0.0 | 1701.0 | 168 | 8 | 2 | 0 | 10 |
| DBX1 | 0.0003 | 7410.0 | 509.1 | 0.0 | 0.0 | 509.1 | 180 | 5 | 0 | 0 | 5 |
| DBX2 | 0.0253 | 110814.8 | 37100.0 | 12005.8 | 530.8 | 49636.6 | 1556 | 274 | 48 | 1 | 323 |
| DBX3 | 0.0071 | 43140.7 | 9487.9 | 4428.0 | 0.0 | 13915.8 | 731 | 74 | 16 | 0 | 90 |
| UNC1 | 0.0033 | 7205.8 | 2205.0 | 1307.4 | 0.0 | 3512.4 | 99 | 17 | 5 | 0 | 22 |
| UNC2 | 0.0098 | 32431.0 | 9098.3 | 4536.8 | 0.0 | 13635.2 | 481 | 67 | 18 | 0 | 85 |
| UMFS | 0.0251 | 119528.1 | 37692.1 | 7707.7 | 2431.1 | 47830.9 | 1841 | 283 | 32 | 5 | 320 |
| NYH1 | 0.0925 | 356712.7 | 118618.5 | 52041.9 | 5331.8 | 175992.2 | 4544 | 860 | 209 | 12 | 1081 |
| NEH1 | 0.0215 | 114844.1 | 34901.5 | 8365.3 | 483.5 | 43750.3 | 1832 | 262 | 35 | 1 | 298 |
| NEH2 | 0.0236 | 114854.7 | 36425.7 | 10184.8 | 1384.6 | 47995.2 | 1751 | 271 | 38 | 3 | 312 |
| MEH2 | 0.0197 | 96663.1 | 31746.3 | 7754.8 | 439.7 | 39940.7 | 1433 | 230 | 30 | 1 | 261 |
